# Pathogens and Molds Affecting Production and Quality of *Cannabis sativa* L.

**DOI:** 10.3389/fpls.2019.01120

**Published:** 2019-10-17

**Authors:** Zamir K. Punja, Danielle Collyer, Cameron Scott, Samantha Lung, Janesse Holmes, Darren Sutton

**Affiliations:** Department of Biological Sciences, Simon Fraser University, Burnaby, BC, Canada

**Keywords:** diseases, plant pathogens, epidemiology, post-harvest molds, fungi, root infection, endophytes

## Abstract

Plant pathogens infecting marijuana (*Cannabis sativa* L.) plants reduce growth of the crop by affecting the roots, crown, and foliage. In addition, fungi (molds) that colonize the inflorescences (buds) during development or after harvest, and which colonize internal tissues as endophytes, can reduce product quality. The pathogens and molds that affect *C. sativa* grown hydroponically indoors (in environmentally controlled growth rooms and greenhouses) and field-grown plants were studied over multiple years of sampling. A PCR-based assay using primers for the internal transcribed spacer region (ITS) of ribosomal DNA confirmed identity of the cultures. Root-infecting pathogens included *Fusarium oxysporum*, *Fusarium solani*, *Fusarium brachygibbosum*, *Pythium dissotocum*, *Pythium myriotylum*, and *Pythium aphanidermatum*, which caused root browning, discoloration of the crown and pith tissues, stunting and yellowing of plants, and in some instances, plant death. On the foliage, powdery mildew, caused by *Golovinomyces cichoracearum*, was the major pathogen observed. On inflorescences, *Penicillium* bud rot (caused by *Penicillium olsonii* and *Penicillium copticola*), *Botrytis* bud rot (*Botrytis cinerea*), and *Fusarium* bud rot (*F. solani*, *F. oxysporum*) were present to varying extents. Endophytic fungi present in crown, stem, and petiole tissues included soil-colonizing and cellulolytic fungi, such as species of *Chaetomium*, *Trametes*, *Trichoderma*, *Penicillium*, and *Fusarium*. Analysis of air samples in indoor growing environments revealed that species of *Penicillium*, *Cladosporium*, *Aspergillus*, *Fusarium*, *Beauveria*, and *Trichoderma* were present. The latter two species were the result of the application of biocontrol products for control of insects and diseases, respectively. Fungal communities present in unpasteurized coconut (coco) fiber growing medium are potential sources of mold contamination on cannabis plants. Swabs taken from greenhouse-grown and indoor buds pre- and post-harvest revealed the presence of *Cladosporium* and up to five species of *Penicillium*, as well as low levels of *Alternaria* species. Mechanical trimming of buds caused an increase in the frequency of *Penicillium* species, presumably by providing entry points through wounds or spreading endophytes from pith tissues. Aerial distribution of pathogen inoculum and mold spores and dissemination through vegetative propagation are important methods of spread, and entry through wound sites on roots, stems, and bud tissues facilitates pathogen establishment on cannabis plants.

## Introduction

*Cannabis sativa* L., a member of the family *Cannabaceae*, is cultivated worldwide as hemp (for fiber, seed, and oil) and marijuana (referred to here as cannabis) (for medicinal and psychotropic effects). The pathogens affecting production of hemp have been described and include fungal, bacterial, viral, and nematode species (McPartland, 1991; McPartland, 1992). In contrast, the pathogens affecting cannabis have not been extensively studied, and the different growing environments, cultivation methods, as well as differences among the strains or genetic selections of hemp and cannabis can influence disease development. This requires that studies on the pathogens potentially affecting cannabis plants be conducted so that methods to manage emerging diseases and molds can be developed. Cannabis plants are propagated from cuttings that are rooted and grown vegetatively, following which they are transferred to conditions of specific reduced lighting regimes (photoperiod) to induce flowering (Small, 2017). Flower buds are harvested, dried, and stored in vacuum-sealed bags or sealed plastic or glass containers prior to distribution. Fungal infection of roots can occur at any time during the production cycle, while colonization of flower buds generally occurs during the later stages of flower development and can be manifested as a pre-harvest or post-harvest bud rot. In addition, foliar pathogens may infect the plant at any stage during its production.

The objectives of this research were to determine the prevalence of root-infecting, foliar-infecting, and flower-infecting fungi affecting cannabis plants grown under indoor environments, in greenhouses, and under field conditions to obtain a better understanding of the diseases affecting this plant. In addition, the incidence of molds in the growing environments, and on pre-harvest and post-harvest inflorescences, was assessed. Cultural methods for isolation, and morphological and molecular methods for identification, were used in this study. More than 22 different fungal and oomycete species and their associated effects on cannabis plants grown indoors and outdoors are presented.

## Materials and Methods

### Isolation of Pathogens and Molds From Cannabis Tissues

**Figure 1:**
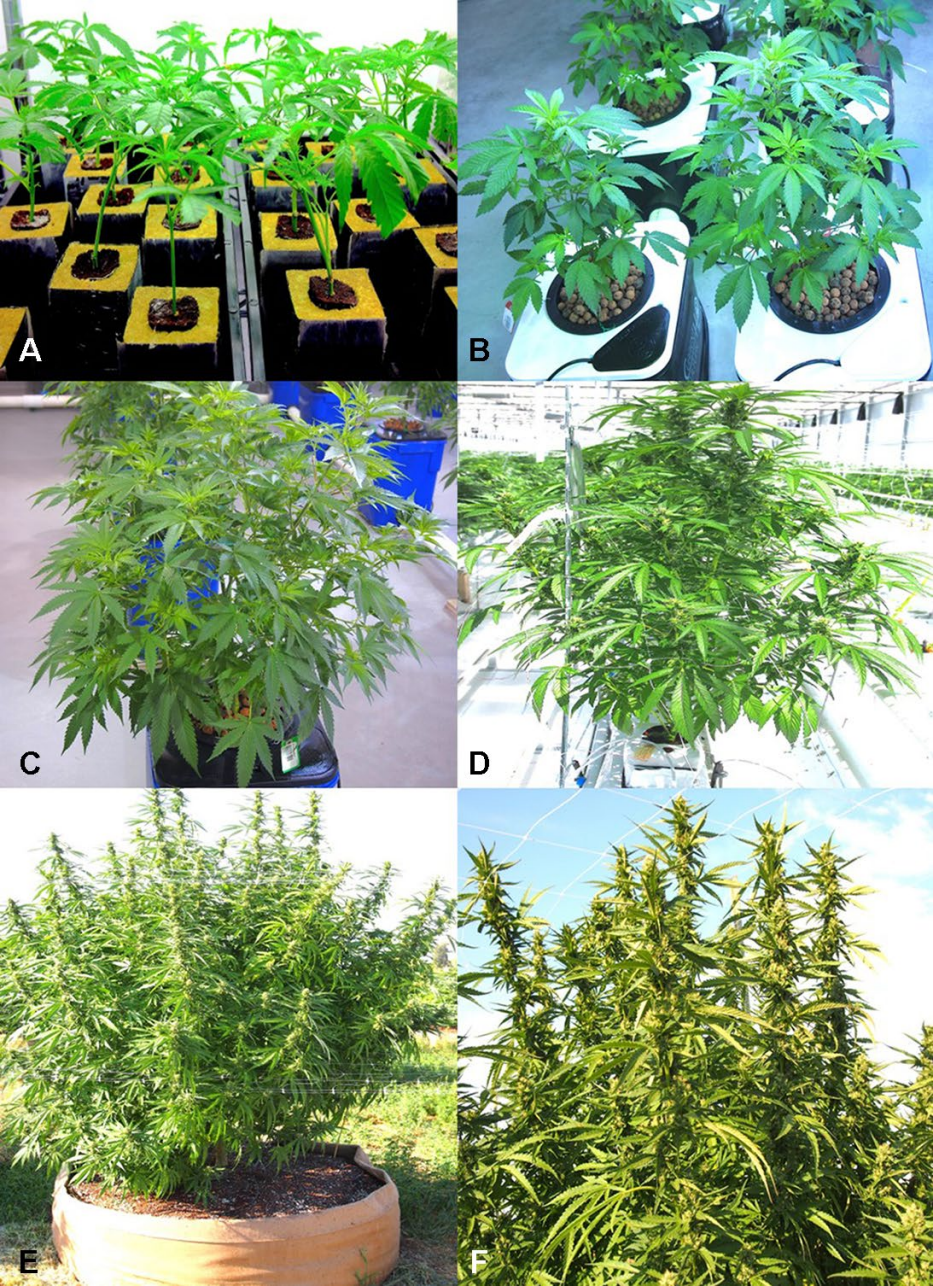
Production systems used for *Cannabis sativa* plants that were sampled in this study. **(A)** Rooting of vegetative cuttings in rockwool plugs containing peat in the central plugs. Cuttings are left for 2 weeks under supplemental lighting to initiate rooting. **(B)** Growth of plants in a hydroponic production system with clay pellets as a substrate. Plants are in the early stages of vegetative development. **(C)** Six-week-old plants in a hydroponic production system ready for transfer from vegetative growth to induction of flowering through controlled photoperiod and light intensity regimes. **(D)** Greenhouse hydroponic production system using coco fiber blocks as a substrate showing a plant in the early stages of flower development. **(E, F)** Field production of *C. sativa* in raised fabric pots under outdoor conditions. **(E)** Plants in early stages of flower development. **(F)** Close-up of shoots bearing flowers. **Figure 1E** reproduced from Can. J. Plant Pathol. 40(4) by permission (Punja et al., 2018).

A range of tissue samples were obtained from cannabis plants grown in indoor controlled environments (two locations) and greenhouse-grown plants (one location) of various cannabis strains (Moby Dick, Hash Plant, Pink Kush, Pennywise, Girl Scout Cookies) under licensed commercial production, as well as from field-grown plants (one location) (**Figure 1**). They included roots, crown tissues, leaves, and flower buds. Samples either displayed symptoms of browning and were presumed to be infected by pathogens or were symptomless. Tissues were sampled at various times during growth of the plants, ranging from early stages of propagation (1–3 weeks old) (**Figures 1A, B**) to advanced vegetative growth (3–6 weeks of age) (**Figures 1C, D**) to plants that were in full flower (7–14 weeks of age) (**Figures 1E, F**). Samples were also obtained of harvested buds before and after they were dried, from indoor and field productions. These tissue samples were obtained over a duration of 3 years, from 2016 to 2018. They were taken at multiple times during the production cycle, and at varying time periods, depending on the pathogen of interest. Each sampling time had a minimum of five replicate samples. All plants were grown indoors and in greenhouses using either Rockwool blocks as a substrate or in coco fiber (coco coir) derived from different commercial suppliers. Plants were watered through an automated irrigation system with individual emitters for each plant. They were provided with the appropriate nutrient regimes and lighting conditions as required for commercial production. A total of around 220 plants were sampled in the study. Among these, around 90 originated from the two indoor production facilities and 120 from the greenhouse facility, all located in British Columbia. In 2019, an additional five samples of diseased tissues were received from one production facility in Ontario showing symptoms of root browning and stem discoloration and five samples of bud tissues originated from a field production site in BC in 2018. Plants with visible symptoms of disease were photographed. Small tissue pieces ca. 0.5 cm in length for roots or 0.2–0.4 cm^2^ for leaves or flower buds were surface-disinfested by dipping them in a 0.5% NaOCl solution for 30 s followed by 20 s in 70% EtOH, rinsed thrice in sterile water, blotted on sterile paper towels, and plated onto Potato Dextrose Agar (PDA, Sigma Chemicals, St. Louis, MO) amended with 100 mg/L of streptomycin sulfate (PDA+S). Dishes containing the tissues were incubated under ambient laboratory conditions (temperature range of 21–24°C with 10–14-h/day fluorescent lighting) for 5–10 days. Emerging colonies were recorded and transferred to fresh PDA+S dishes for subsequent identification to the genus level using morphological criteria, including colony color and size and microscopic examination of spores. Species-level identification was done by PCR using the primers ITS1F-ITS4 (ITS1-F 5’-CTTGGTCATTTAGAGGAAGTAA-3’ and ITS4 5’-TCCTCCGCTTATTGATATGC-3’). The resulting sequences were compared to the corresponding ITS1-5.8S-ITS2 sequences from the National Center for Biotechnology Information (NCBI) GenBank database to confirm species identity using only sequence identity values above 99%. These sequences have been deposited in GenBank. Pathogenicity tests were conducted for representative isolates (a minimum of two) of *Fusarium oxysporum* and *Fusarium solani* recovered from roots and two isolates each of *Botrytis cinerea* and *Penicillium olsonii* recovered from flower buds, following the methods described by Punja and Rodriguez (2018) and Punja (2018).

### Scanning Electron Microscopy

Powdery mildew infection of leaves, and infection of buds by *P. olsonii* and *F. oxysporum* following artificial inoculation with spores, as well as stem segments showing pith tissues, was prepared for scanning electron microscopy as follows. Tissue segments ca. 0.5 cm^2^ were adhered to a stub using a graphite-water colloidal mixture (G303 Colloidal Graphite, Agar Scientific, UK) and Tissue-Tek (O.C.T. Compound, Sakura Finetek, NL). The sample was submerged in a nitrogen slush for 10–20 s to rapidly freeze it. After freezing, the sample was placed in the preparation chamber of a Quorum PP3010T cryosystem attached to a FEI Helios NanoLab 650 scanning electron microscope (Dept. of Chemistry, 4D Labs, Simon Fraser University). The frozen sample was sublimed for 5 min at −80°C, after which a thin layer of platinum (10-nm thickness) was sputter-coated onto the sample for 30 s at a current of 10 mA. The sample was moved into the SEM chamber, and the electron beam was set to a current of 50 pA at 3 kV. Images were captured at a working distance of 4 mm, at a scanning resolution of 3072 x 2207 collected over 128 low-dose scanning passes with drift correction.

### Mold Sampling in Different Growing Environments

To assess the potential for airborne dispersal of mold and pathogen spores within different growing environments, 9-cm diameter petri dishes containing PDA+S were placed with the lids removed on benches in areas between rows of plants, at approximately 1-meter intervals, in both indoor growing environments and in the greenhouse during 2018. Field sampling was not conducted. The dishes were left for 60 min and then lids replaced and brought back to the laboratory. All air sampling was done during the period of 11:00–13:00 h. A minimum of 12 replicate dishes was included at each sampling location. Control dishes were placed in similar locations with the petri dish lids left on. Fungal colonies that developed after 5–7 days were counted, and representative ones were subcultured for identification. The sampling was repeated in two different indoor environments at various time periods (March–September) during 2018 and repeated three times within one greenhouse facility. In the indoor facilities, the sampling was conducted weekly in the same growing room over 6 sequential weeks (June–July 2018) to assess changes in the mold populations over time. In the greenhouse facility, the sampling was repeated weekly over 4 weeks (June–September 2018). The sampling time was kept the same in all studies. Fungal colonies were identified to genus level using morphological criteria. Specific colonies were subcultured onto fresh medium and used for DNA extraction. Molecular identification to genus and species level was conducted as described previously. Mean colony-forming units of each fungal genus per petri dish was determined, and standard error of the means was calculated from the replications and repetitions.

### Isolation of Fungi From Coco Fiber Substrates

Samples consisting of approximately 5–10 g of coco fiber (coco coir) substrate used for growing plants were obtained at multiple times during the production cycle in five indoor and greenhouse facilities to assess the diversity and total populations of fungi present. In addition, samples were taken from previously unopened and unused bags. The brand names included Mo’KoKo, Royal Gold (Humboldt County, CA), Canna Coco (Toronto, Canada), Forteco, and Rio (Irving, TX). A subsample of 0.5 g was suspended in 10 ml of sterile distilled water and vortexed for 20 s. A 1-ml suspension was transferred to 9 ml of water, shaken, and a further dilution was made in 9 ml of water. Aliquots (0.5 ml) of each suspension were streaked onto two replicate PDA +S plates and repeated three times for each sample. The plates were incubated for 5–7 days under ambient laboratory conditions and then examined for diversity and numbers of microbes present. Fungal colonies were identified to genus level where possible using morphological criteria. Specific colonies were subcultured onto fresh medium and used for DNA extraction and molecular identification as described previously.

### Isolation of Fungi From Internal Tissues of Plants

The presence of naturally occurring endophytic fungi within stem tissues of *C. sativa* “Moby Dick” plants was determined through dissection of a mature indoor-grown plant grown using coco fiber (Canna Coco) as a substrate. Plants were provided with 24 hr of light through an Agrobrite T5H0 Fixture (Hydrofarm Inc., Petaluma, CA) containing four 6,400K spectrum bulbs with a light intensity of 9,400 lumens to maintain vegetative growth. The temperature range was 23–28°C. Fertilization was achieved through a mixture of Advanced Nutrients: pH Perfect Sensi Grow A and B and CALiMAGic by General Hydroponics (Sebastopol, CA) each at a rate of 1 ml/L (pH 5.8). Plants were watered approximately once a day until runoff. The main stem of the plant was sectioned into 5-cm long segments, beginning at the crown and proceeding to the top of the plant through two lateral branches on each side, a distance of around 75 cm. The stem pieces were surface-sterilized in a 10% bleach solution (Javex, containing 6.25% NaOCl) for 20 s followed by 70% EtOH for 20 s and rinsed with sterile distilled water for 1 min. The segments were transferred to a sterile petri dish, where they were cut lengthwise with a scalpel and small tissue pieces, measuring approximately 0.5 cm^2^ were cut to represent the cortex/vascular tissues and the pith, which were plated separately. Thinner stem pieces included just the vascular and cortical tissues without the pith. A total of four tissue pieces of each type were placed onto each of two petri dishes containing PDA+S and incubated under ambient laboratory conditions for 1 week before microbial presence was assessed. In the next series of experiments, three additional strains of *C. sativa* were used to establish the extent of internal colonization by microbes. These strains were “Pennywise,” “Space Queen,” and “Cheesequake.” Tissue segments representing stem pieces, petioles, and nodal segments (approx. 0.5 mm in length) were excised from plants grown as described above and surface-sterilized in a 10% bleach solution for 1 min, followed by 70% EtOH for 30 s and then rinsed in sterile distilled water for 1 min and plated onto PDA+S dishes. The number of fungal colonies emerging from the tissue pieces was recorded, and the genera were identified by morphological examination of the colony or spore type. Molecular confirmation was conducted as described previously for selected cultures. Bacteria and yeasts were excluded from the total counts of microbial presence. The experiment was conducted twice using different plants of the same strains.

### Endophytic Colonization of Stem Tissues

Plants of *C. sativa* L. were grown in coco fiber as a substrate under a 200-watt Sunblaster CFL light and fertilized as described previously. The uppermost 2 cm growing region of the plant (at 65-cm distance from the crown) were cut; 1-cm long segments were removed from just below the cut end and then surface-sterilized in 10% bleach for 1 min, followed by 70% EtOH for 30 s and then rinsed in sterile distilled water for 1 min. Pieces measuring 0.5 cm in length were placed on PDA+S (300 mg/L). This procedure was conducted to check for presence of background endophytes. The wounded exposed stem surfaces on the plant (with eight replicates) were then inoculated by placing a mycelial plug (approx. 1 cm^2^) on the surface of the cut stem (mycelial side down) and left in place for 7 days. Controls received a PDA plug or were left uninoculated. Cultures of the fungi used were grown on PDA+S for 2 weeks before being used. The fungi tested were recovered from internal tissues of cannabis plants as described in the preceding section. They were identified as *Chaetomium globosum*, *F. oxysporum*, *P. olsonii*, *Trametes (Polyporus) versicolor*, and *Trichoderma harzianum*. After 7 days, the plug was removed, and stem segments were excised at distances of 1, 3, and 6 cm below the initial cut site that was inoculated with the plug. These segments were surface-sterilized as described previously and plated on PDA+S (300 mg/L). The colonization of each stem segment by each of the respective fungi at each distance was rated after 7 days. The experiment was conducted twice. The data was expressed as means +/− standard deviations.

### Mold Sampling on Bud Tissues

Mold assessments on pre-harvest and post-harvest flower buds were made using a cotton swab procedure during 2017–2018. Sterile cotton swabs were gently wiped across the surface of buds on plants either prior to harvest or following harvest, as well as at various stages of a mechanized trim operation that removed bract and leaf tissues surrounding the inflorescence. This was repeated from replicate samples at multiple time periods in two different facilities. The swabs were streaked across a PDA+S dish which was then brought back to the laboratory and incubated under ambient conditions as described previously. The swab method was also used to assess the presence of fungi on freshly cut and healed stems on cannabis plants following regular pruning of shoots in both an indoor and greenhouse growing facility. For harvested dried buds, small segments ca. 2 mm were taken from replicate samples (total of 50) at multiple time periods (up to 8) and were placed directly onto PDA+S dishes, or following a 20 s dip in 70% EtOH. Following incubation for 7 days under ambient laboratory conditions, enumeration of fungal colonies on the dishes (bacterial colonies were excluded) was conducted; representative morphologically unique colonies were subcultured onto fresh PDA+S dishes and used for DNA extraction and PCR-ITS identification to species level as described previously.

## Results

### Isolation of Pathogens and Molds From Cannabis Tissues

**Figure 2:**
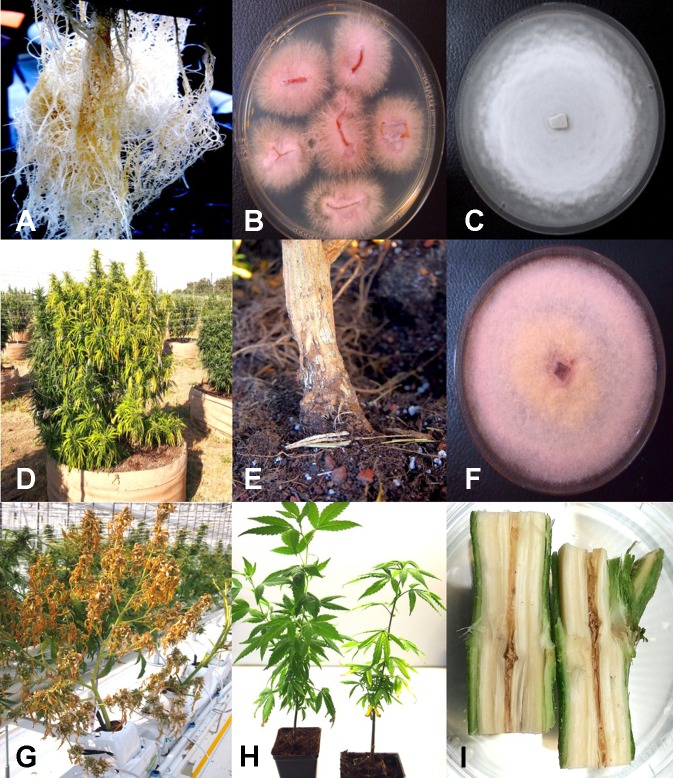
Root-infecting pathogens on *Cannabis sativa*. **(A)** Symptoms of brown discoloration on the root system of indoor hydroponically grown plants. **(B)** Colonies of *Fusarium oxysporum* isolated from diseased roots in **(A)** growing on potato dextrose agar. **(C)** Colony of *Pythium catenulatum* isolated from diseased roots growing on potato dextrose agar. **(D)** Symptoms of natural crown infection on a field-grown cannabis plant caused by a combination of *F. oxysporum*, *Fusarium brachygibbosum*, and *Pythium aphanidermatum*. **(E)** The crown area of the infected plant shown in **(D)** is sunken, and there is visible mycelial growth on the surface. **(F)** Colony of *Fusarium brachygibbosum* isolated from diseased roots growing on potato dextrose agar. **(G)** Symptoms of plant collapse as a result of infection by *P. aphanidermatum* under a greenhouse environment. **(H)** Comparison of a noninoculated plant (left) with a plant wound-inoculated with spores of *F. oxysporum* (right) and grown in coco fiber substrate. Photo was taken 4 weeks after inoculation and shows stunting and yellowing of leaves. **(I)** Symptom of internal discoloration of the pith tissue in the upper 10 cm of the crown region of a plant grown indoors in coco fiber as a substrate and infected by *F. oxysporum*. **Figures 2A, D, E, G** reproduced from Can. J. Plant Pathol. 40(4) by permission.

From cannabis plants grown in an indoor hydroponic production system in which brown roots were visible (**Figure 2A**) and from a greenhouse production system in which coco fiber was used as a growing substrate and with visible brown roots, samples were collected and used for isolation. Colonies of *F. oxysporum* (**Figure 2B**) and *Pythium* species that included *Pythium dissotocum*, *Pythium myriotylum*, *Pythium aphanidermatum*, *Pythium ultimum*, and *Pythium catenulatum* (**Figure 2C**) were recovered and identified based on ITS 1-ITS2 rDNA sequence comparisons to GenBank. Additional species of *Fusarium* that have been recovered from diseased cannabis root and crown tissues include *F. solani* and *Fusarium proliferatum*. From tissue samples originating from Ontario, *F. oxysporum*, *P. myriotylum*, and *P. dissotocum* were recovered from symptomatic crown and root tissues. From field-grown plants with symptoms of yellowing foliage (**Figure 2D**) and sunken lesions present on the crown of affected plants (**Figure 2E**), *F. oxysporum*, *P. aphanidermatum*, and *Fusarium brachygibbosum* (**Figure 2F**) were isolated and identified. From a greenhouse-grown plant close to harvest and displaying symptoms of browning and plant collapse (**Figure 2G**), *P. aphanidermatum* was isolated. The pathogenicity of two isolates of *F. oxysporum* and *F. solani* originating from cannabis plants was confirmed by re-inoculation of rooted cannabis cuttings. The results from inoculation with *F. oxysporum* are shown in **Figure 2H**, in which symptoms of stunting and yellowing were apparent after 3–4 weeks. The pith tissues of these plants exhibited browning (**Figure 2I**), and the pathogen was reisolated. For the *F. solani* isolates tested, similar symptoms were observed, except that root and pith browning were more extensive. Therefore, individual root pathogens as well as combinations of pathogens may be recovered from symptomatic cannabis plants grown indoors and under field conditions.

**Figure 3:**
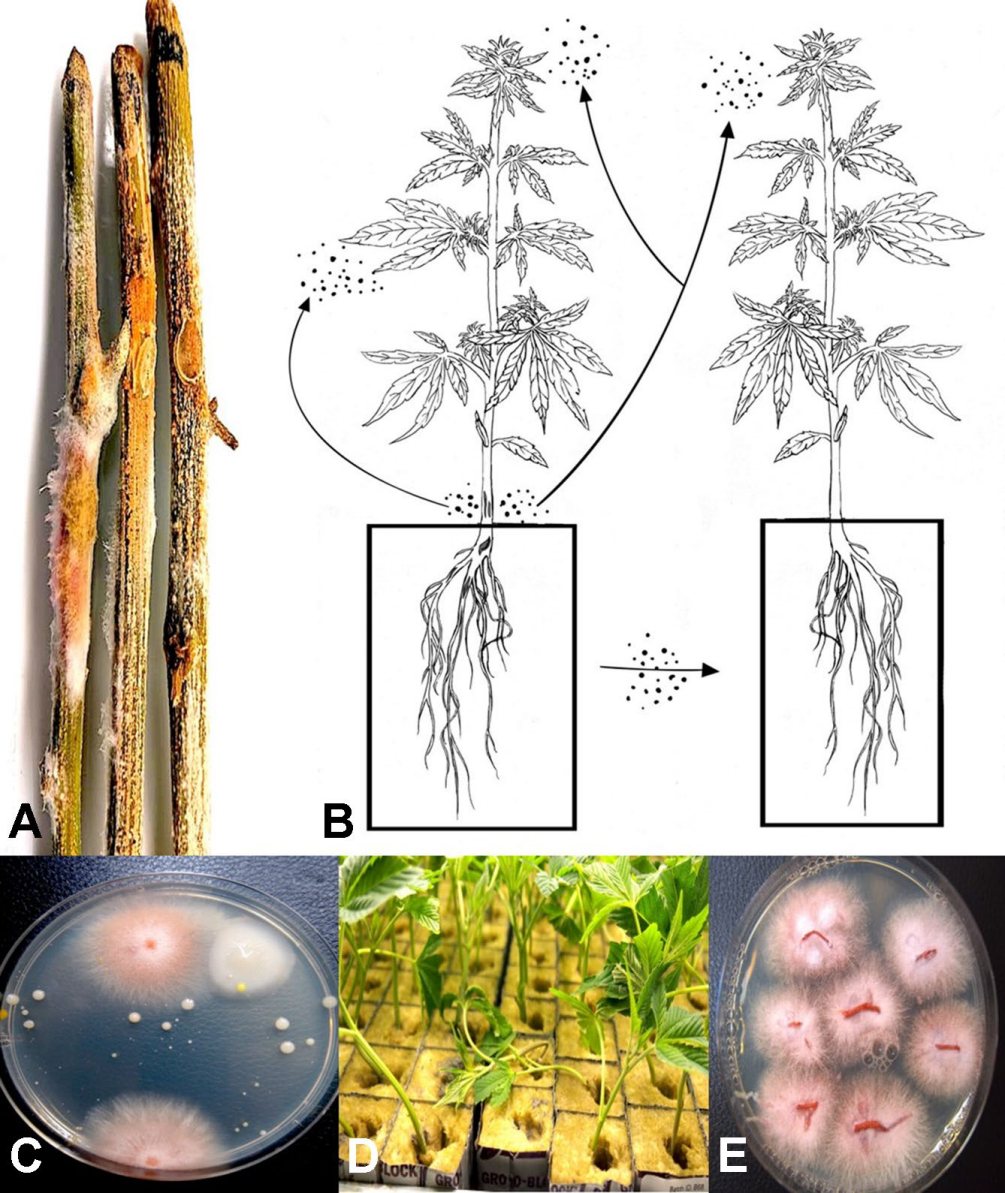
**(A)** Mycelial growth and sporulation of *Fusarium* species on cannabis stems. **(B)** Schematic diagram showing the potential for spread of spores of *Fusarium* from stem tissues to leaves and flower buds of the same and adjacent plants. As well, spread can occur to adjoining plants by water. **(C)** Colonies of *Fusarium oxysporum* detected in hydroponic nutrient solution following plating of samples onto potato dextrose agar + streptomycin sulfate. **(D)** Damping-off on cuttings of cannabis in rockwool blocks resulting from spread of *F. oxysporum* and infection of the cut ends of the stem. **(E)** Colonies of *F. oxysporum* isolated from roots and stems of infected cuttings shown in **(D)**. **Figure 3A** reproduced from Can. J. Plant Pathol. 40(4) by permission.

The potential for production of spores of *Fusarium* species on stem tissues of cannabis plants was demonstrated by inoculating mycelial plugs onto harvested stem segments and incubating them under high humidity conditions for 5 days. Prolific spore production, which can result in spread of inoculum into the air, can potentially result in foliar or flower bud infection on the same or adjacent plants (**Figure 3A**). In addition, spores of *F. oxysporum* may be spread though water or hydroponic nutrient solution as demonstrated by recovery on PDA (**Figure 3B**), and if recirculated without treatment to destroy pathogen spores (**Figure 3C**), it can introduce inoculum into propagation rooms where cuttings are being rooted, causing mortality (**Figure 3D**) and crown and root infection from which *F. oxysporum* was readily isolated (**Figure 3E**). Therefore, *F. oxysporum* is capable of infecting at multiple locations within a production facility.

**Figure 4:**
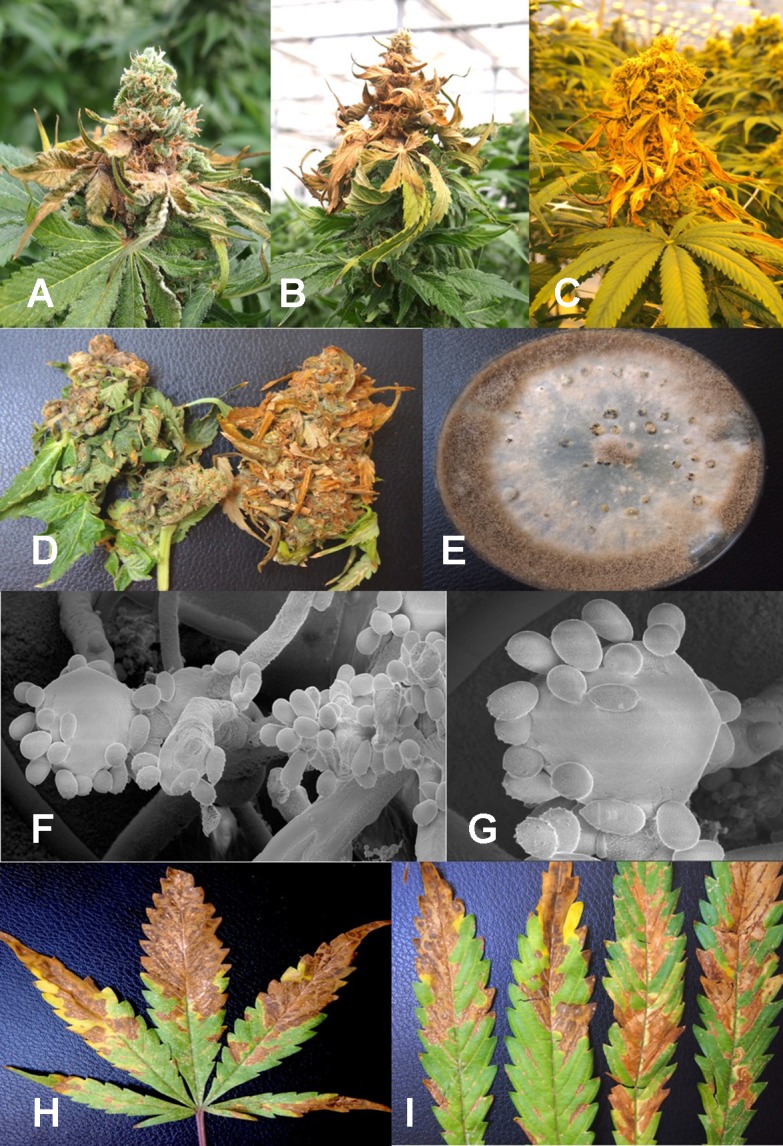
*Botrytis* bud rot development, caused by *Botrytis cinerea*, in a greenhouse production facility. **(A)** Early infection on developing inflorescence, showing browning and decay of leaves and bracts. **(B, C)** Advanced stages of bud rot, where the entire inflorescence has been destroyed. **(D)** Close-up of diseased harvested inflorescences, showing development of mycelium within the bud and decay. **(E)** Colony of *B. cinerea* recovered from diseased tissues showing prolific sporulation on the edge of the colony and sclerotial development in the center. **(F, G)** Scanning electron micrographs of conidiophores and conidia of *B. cinerea* from culture. The points of spore attachment to the conidiophore head can be seen. **(H, I)** Lesions on cannabis leaves resulting from spore deposition of *B. cinerea* from infected inflorescences to cause individual spots that enlarged into necrotic lesions.

From flower buds with symptoms of brown discoloration, blighting of bracts and leaves and decay of the tissues (**Figures 4A–C**), grayish-brown mycelium was observed when the tissues were incubated in a plastic bag for 48 hr (**Figure 4D**), and colonies recovered with gray sporulation were identified as *B. cinerea* causing bud rot (**Figure 4E**). Spores were formed on conidiophores and borne in clusters (**Figures 4F, G**). In severe cases of disease incidence (up to 50% of plants affected), leaves on cannabis plants with bud rot also displayed leaf lesions (**Figures 4H, I**). The lesions developed as small circular spots which enlarged to coalesce into necrotic areas that were sometimes surrounded by yellow margins and in many cases delimited by the leaf veins. Surface-sterilized tissue pieces plated onto PDA+S yielded colonies similar to those shown in **Figure 4E**. These foliar infections due to *B. cinerea* have not been previously reported on cannabis plants and appear to occur only under conditions of high inoculum levels and on plants approaching harvest.

**Figure 5:**
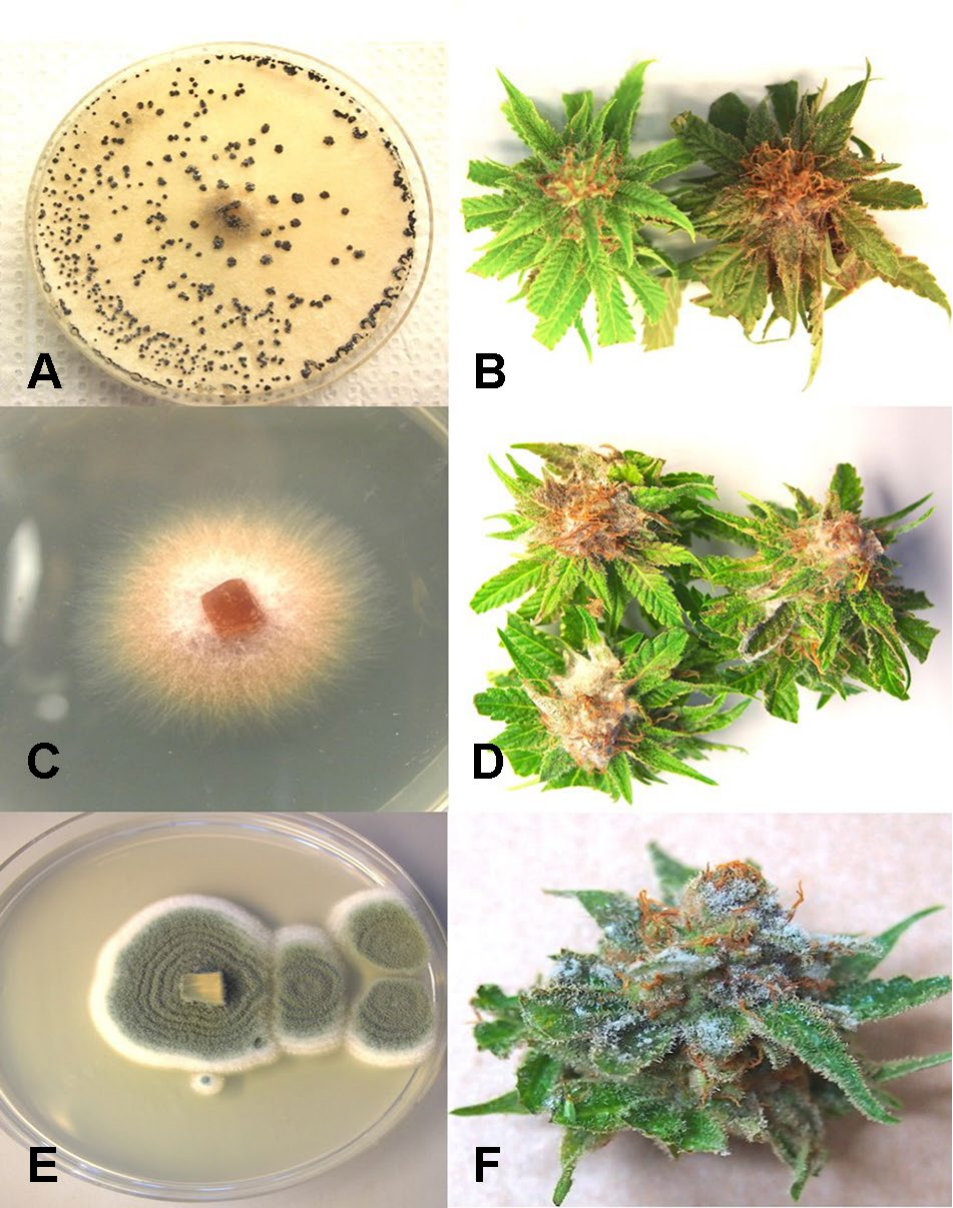
Inoculation experiments conducted on developing buds of *Cannabis sativa* to determine the extent of disease development caused by 3 fungi. **(A, B)** Inoculation with *Botrytis cinerea* and culture morphology of the *B. cinerea* isolate used. **(C, D)** Inoculation with *Fusarium oxysporum* and colony morphology of the isolate of *F. oxysporum* used. **(E, F)** Inoculation with *Penicillium olsonii* and colony morphology of the *P. olsonii* isolate used.

From samples of 50 harvested flower buds that were fresh or had previously been dried, three fungal species were identified: *B. cinerea* (**Figure 5A**), *F. oxysporum* (**Figure 5C**), and *P. olsonii* (**Figure 5E**). The overall frequencies of recovery were 2, 2.7, and 7.4%, respectively. When these fungi were inoculated onto fresh flower buds and incubated under conditions of high humidity, all of them were capable of causing browning of the tissues and decay to varying extents (**Figures 5B, D, F**).

### Scanning Electron Microscopy

**Figure 6:**
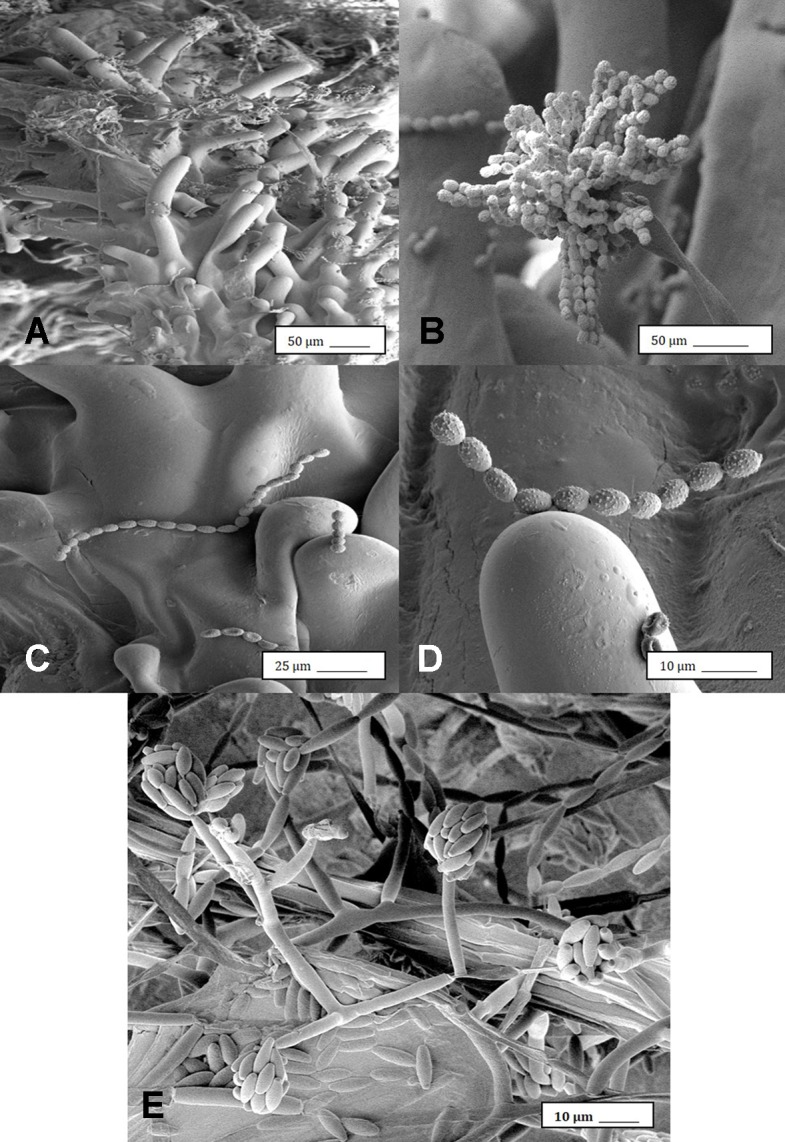
Scanning electron microscopic images of the development of mycelium and spore production by *Penicillium olsonii* on inoculated cannabis inflorescences. **(A)** Spores and mycelium on stigmatic hairs (papillae). **(B)** Conidiophore with chains of conidia characteristic of *Penicillium* formed on the bud surface. **(C, D)** Close-up views of spore chains of *P. olsonii* stuck to stigmatic hairs. **(E)** Conidiophore and conidia of *Fusarium oxysporum* on inoculated flower bud.

**Figure 7:**
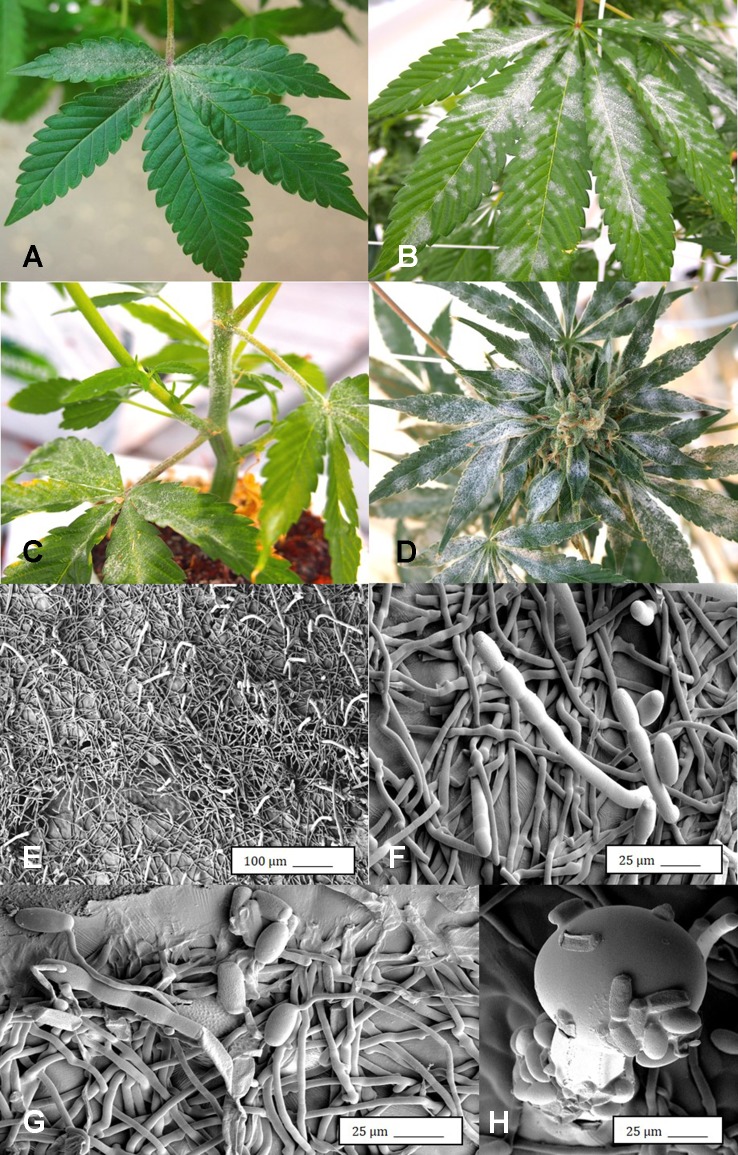
Powdery mildew development on leaves, stems, and flower buds of *Cannabis sativa*, caused by *Golovinomyces cichoracearum*. **(A)** Early stages of infection on young leaf, showing sparse white mycelium on leaf surface. **(B)** Advanced stages of infection with profuse sporulation, resulting in a powdery appearance on the leaf surface. **(C)** Development of powdery mildew on leaves and stem of vegetative cuttings of strain “Pink Kush.” **(D)** Powdery mildew infection on inflorescences of *C. sativa* “Pink Kush” showing extensive mycelial development. **(F)** Scanning electron micrograph of mycelium and spores produced on conidiophores developing on the surface of heavily infected leaves. **(G)** A close-up view of conidiophores with a chain of powdery mildew spores attached. **(H)** Germination of spores to produce a mycelial network on the leaf surface. **(I)** Spores of G. *cichoracearum* adhering to the surface of a glandular trichome on the surface of a leaf bract.

Under the scanning electron microscope, cannabis flower buds that had been inoculated with a spore suspension of *P. olsonii* showed the presence of abundant mycelial growth and sporulation on the stigmatic surface (**Figures 6A, B**), and chains of spores were formed that were stuck to the stigmatic hairs (papillae) (**Figures 6C, D**). Similarly, flower buds inoculated with a spore suspension of *F. oxysporum* also showed abundant pathogen sporulation (**Figure 6E**). Leaves with natural infection by powdery mildew initially showed white mycelial growth, followed by abundant sporulation of the pathogen which caused the leaves to develop a white powdery appearance (**Figures 7A, B**). In addition, infection was observed on stems (**Figure 7C**) and on inflorescences (**Figure 7D**). Under the scanning electron microscope, abundant mycelial growth on the leaf surface was accompanied by spores that were produced on conidiophores and were borne in chains (**Figures 7E–G** **)**. Spores were also observed to germinate on the leaf surface (**Figure 7H**), and they were found adhered to the surface of glandular trichomes (**Figure 7I**). The pathogen was identified by ITS1-ITS2 rDNA sequence comparisons available in GenBank as *Golovinomyces cichoracearum*. However, isolates from cannabis could not be distinguished using the ITS region from *Golovinomyces ambrosiae* reported to infect sunflower and giant ragweed and *Golovinomyces spadiceus* from dahlia (Punja, 2018). Therefore, the species of powdery mildew affecting cannabis is provisionally named *G. cichoracearum sensu lato* and will require additional sequence comparisons of gene regions other than the ITS to confirm the species identity.

### Mold Sampling in Different Growing Environments

**Figure 8:**
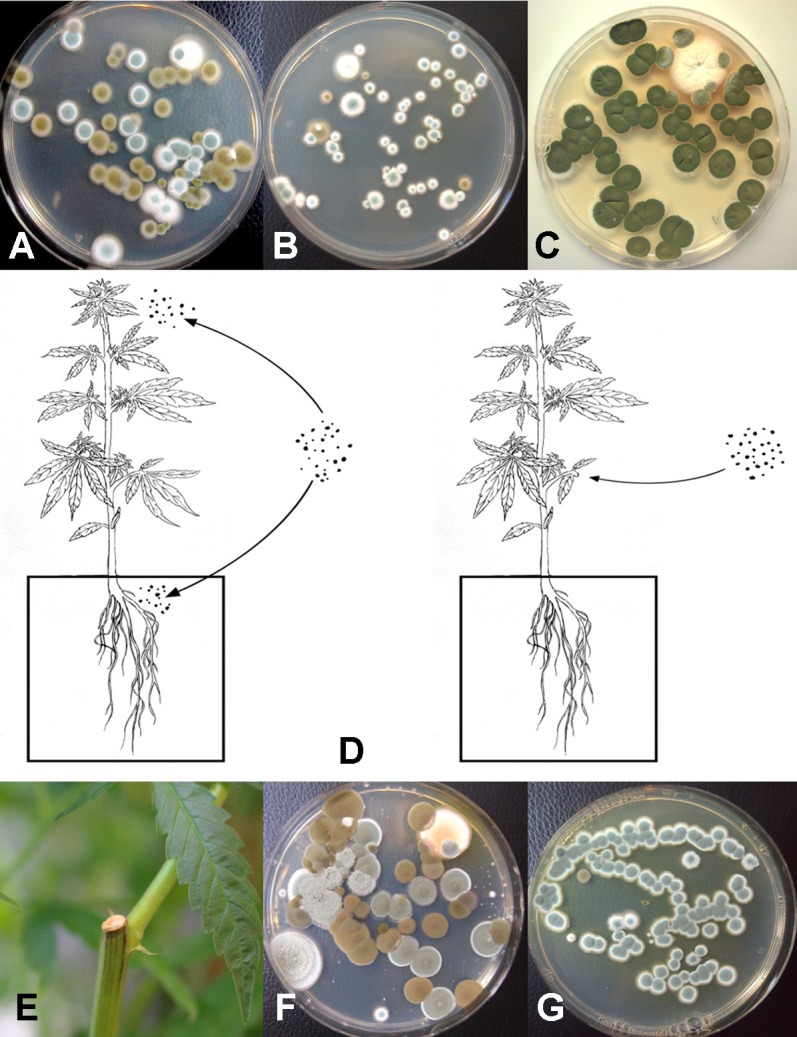
Aerial spore dispersal of molds in the growing environment of cannabis plants. All petri dishes contain potato dextrose agar with 100 mg/L of streptomycin sulfate (PDA+S). **(A)** Petri dishes were exposed for 60 min in a greenhouse environment or **(B)** in an indoor environment and incubated under laboratory conditions for 5 days. Both *Cladosporium* (brown to black colonies) and *Penicillium* (blue-green colonies) were observed growing on the dishes. **(C)** Petri dishes exposed outdoors showed primarily the growth of *Cladosporium* colonies. **(D)** Proposed scheme through which air-borne spores can affect quality of cannabis plants. Air-borne spores may establish in the substrate, on inflorescences, or on the cut exposed surface of pruning wounds. **(E)** Cut surface following pruning of a stem which was swabbed and streaked onto PDA. **(F)** Colonies of *Penicillium* and *Cladosporium* growing from a swab taken off a pruning site on the stem of a greenhouse-grown plant. **(G)** Colonies of *Penicillium* emerging from a swab taken off a pruning site on the stem of an indoor-grown plant and streaked onto PDA.

The placement of petri dishes containing potato dextrose agar plus 100 mg/L of streptomycin sulfate with the lids removed for periods of up to 1 h in greenhouses and indoor controlled environment growing facilities of cannabis provided an indication of the types of molds that were present within each growing environment. Under greenhouse conditions, the principal mold genera recovered were *Cladosporium* and *Penicillium* (**Figure 8A**). In indoor growing environments, *Penicillium* species were most prevalent (**Figure 8B**). The potential sources of these fungi are from decaying plant material, growing substrates used such as coco fiber, as well as indoor structures and equipment. By comparison, petri dishes placed in greenhouse environments showed a high level of *Cladosporium* (**Figure 8C**). Once airborne, the spores can land on leaves, flower buds, cut exposed stems, or growing substrates such as Rockwool and colonize the substrate (**Figure 8D**). The cut surfaces of stems that had been pruned (**Figure 8E**) and were forming wound response tissue yielded both *Cladosporium* and *Penicillium* species from a greenhouse environment (**Figure 8F**), similar to those found in air samples (**Figure 8A**). On indoor plants where cut stems were sampled, *Penicillium* species, as well as *F. oxysporum*, were recovered (**Figure 8G**).

**Figure 9:**
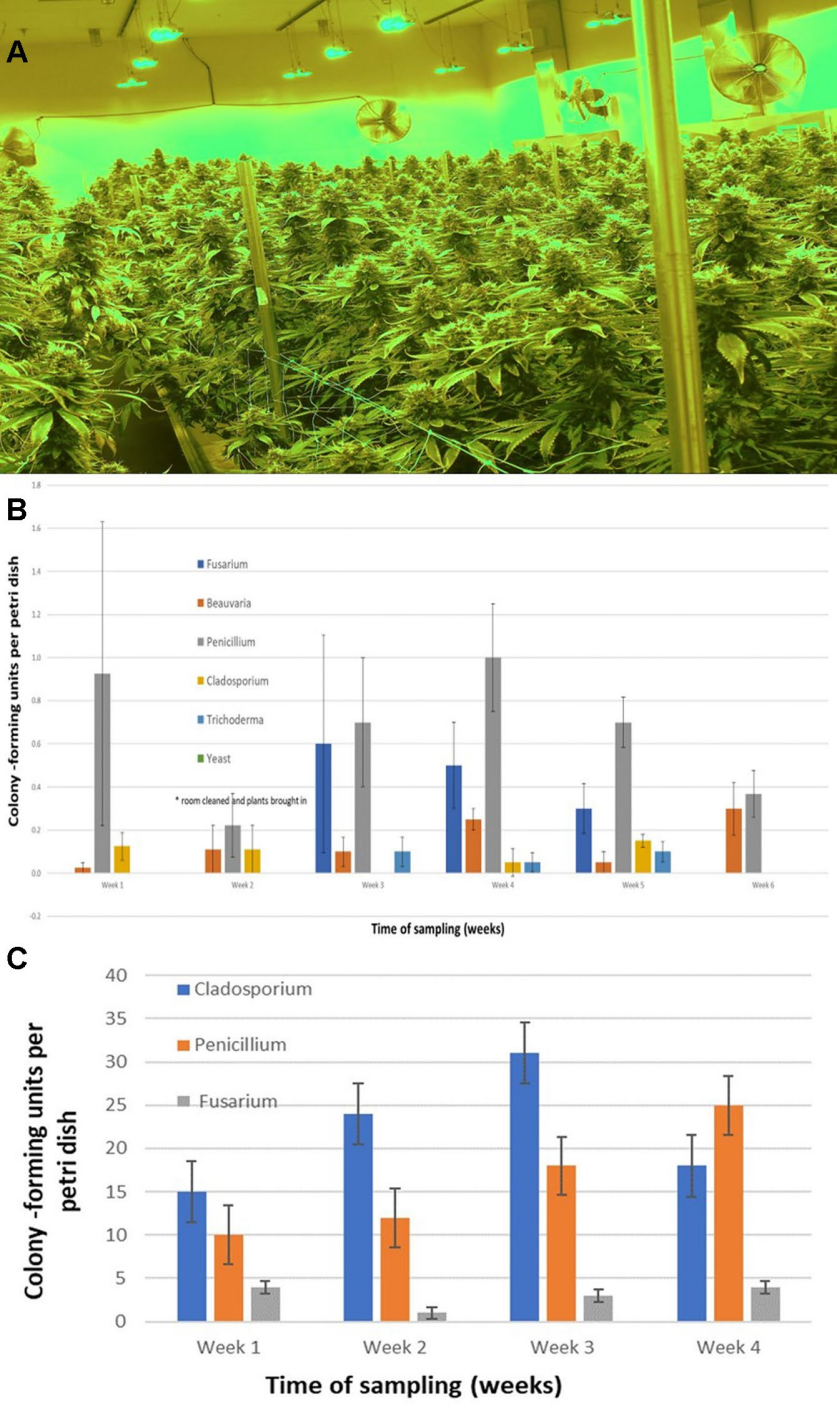
**(A)** Cannabis plants growing in an indoor facility close to harvest. **(B)** Quantification of molds present in air samples in an indoor growing facility over a 6-week period. In week 2, plants were established in the growing room. Petri dishes containing PDA+S were exposed for 60 min to ambient conditions and brought back to the laboratory. Colony-forming units of fungal species were assessed after 5–7 days. The five fungal general present are indicated. Data are means +/− standard errors from 12 replicate dishes. **(C)** Quantification of molds present in air samples in a greenhouse growing facility over a 4-week period, from the time plants were established in week 1. Petri dishes containing PDA+S were exposed for 60 min to ambient conditions and brought back to the laboratory. Colony-forming units of fungal species were assessed after 5–7 days. The three main fungal genera present are indicated. A few colonies of *Aspergillus* and *Epicoccum* were also observed (data not shown). Data are the means +/− standard errors from three repeated experiments.

The air sampling procedure using exposed petri dishes was conducted over a 6-week period in an indoor controlled environment growing facility (**Figures 9A, B**) as well as over a 4-week period in a greenhouse facility (**Figure 9C**). The results showed several relevant findings: (i) Following a thorough cleaning of the indoor facility, which showed high levels of *Penicillium* species (in week 1), mold levels were initially very low in week 2 when plants were introduced (with *Beauveria bassiana*, *P. olsonii*, and *Cladosporium westerdijkieae* present at low background levels). (ii) *Fusarium oxysporum* and *Penicillium* population levels increased following the introduction of cannabis plants in week 3, and *T. harzianum* was detected (**Figure 9B**). (iii) The population levels of the fungal species were variable in weeks 4–6, with *Penicillium* representing the most frequently detected mold. The presence of *B. bassiana* and *T. harzianum*, both of which are registered as biological control agents (BotaniGard and RootShield, respectively) and had been applied within the facility for control of thrips and *Fusarium* root rot in the week preceding sampling, was interesting to see as a component of the air-borne mold population. (iv) In the final week of sampling (week 6), the predominant fungi found were *Beauveria* and *Penicillium*, and no *Fusarium* was detected. In the greenhouse facility, a similar air sampling study conducted over a 4-week period showed that the predominant fungi found were *Cladosporium*, *Penicillium*, and low levels of *Fusarium* (**Figure 9C**); however, the total colony-forming units were higher in the greenhouse facility (maximum of 30 cfu/petri dish) compared to those found in the indoor growing environment (maximum of 1 cfu/petri dish).

### Isolation of Fungi From Coco Fiber Substrates

**Figure 10:**
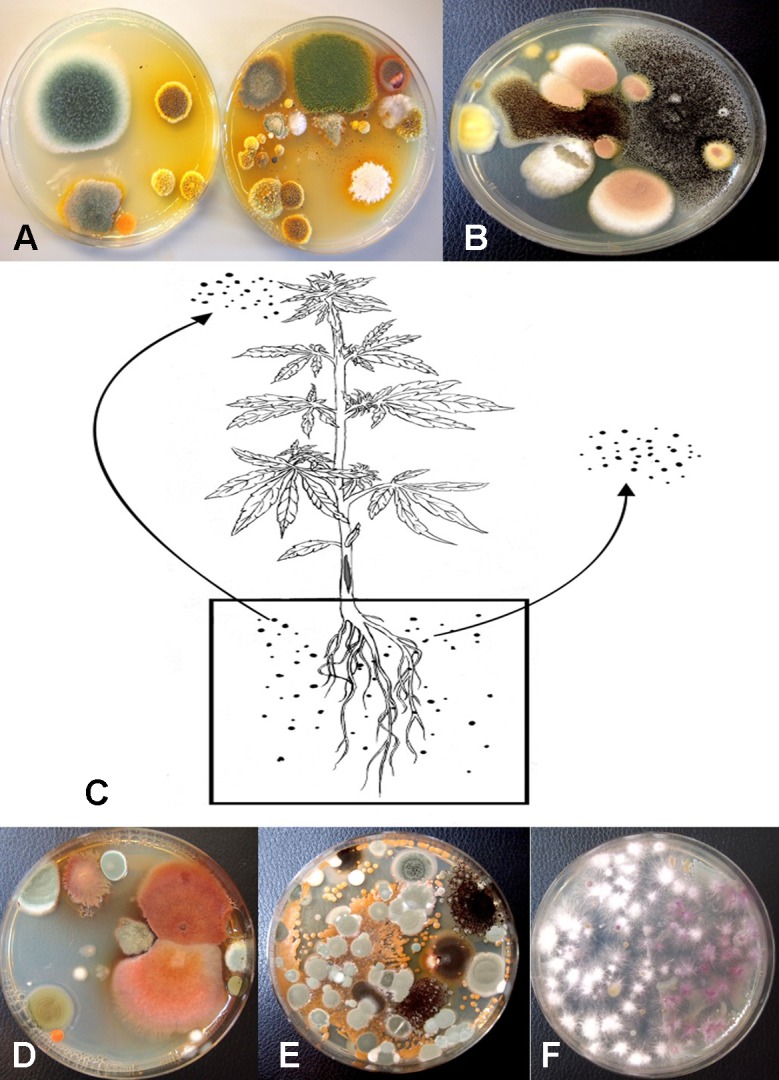
Recovery of fungal species from samples of unsterilized coconut fiber (coco) used in the hydroponic cultivation of cannabis plants. Samples were diluted in water and plated onto PDA+S. **(A)** A diverse range of *Penicillium* and *Aspergillus* species were recovered from unused coco bags. **(B)** Colonies of *Aspergillus niger* (black) and *Aspergillus terreus* (pink) present in coco samples. **(C)** Proposed scheme through which molds found in growing substrates could be air-borne and spread to the inflorescences, or grow internally in the pith tissues of the stem. **(D, E)** Microbes present in coco substrate at the beginning and end of the production cycle include species of *Aspergillus* (red colonies), *Penicillium* (blue-green colonies) as well as a range of uncharacterized bacteria. **(F)** Colonies of *Fusarium oxysporum* emerging from coco substrate used in cannabis production, showing complete colonization of the medium by the end of the 10-week production cycle as a result of build-up of inoculum.

Following serial dilution and plating of samples of coco fiber onto PDA+S Petri dishes, a large and diverse number of fungi, yeast and bacteria were observed growing after 5 days of incubation (**Figures 10A, B**). The range of fungi identified included *P. olsonii* and *Penicillium chrysogenum*, *Aspergillus niger*, *Aspergillus ochraceus*, *Aspergillus terreus*, *Rhizopus stolonifer*, *T. harzianum*, *B. bassiana*, *F. oxysporum*, and other unidentified species. All of these, especially *A. niger* and *Penicillium* species, were present in unopened bags originating from different sources. The potential for spread of spores of these fungi as air-borne propagules during cultivation of plants to leaves and flower buds of cannabis plants is possible (**Figure 10C**). Not all coco fiber substrates tested were contaminated to a similar level with these fungi, and some products (which had been sterilized) were mostly found to contain only *Penicillium* species (data not shown). The extent to which coco fiber substrates harbored total microbial populations increased over time of usage for plant growth, and at the end of the cropping cycle, the populations of bacteria and yeast were considerably higher than fungal populations (**Figure 10D** compared to **Figure 10E**). In some samples, *F. oxysporum* was the most predominant microbe in the end-of-cycle coco fiber samples (**Figure 10F**).

### Isolation of Fungi From Internal Tissues of Plants

**Figure 11:**
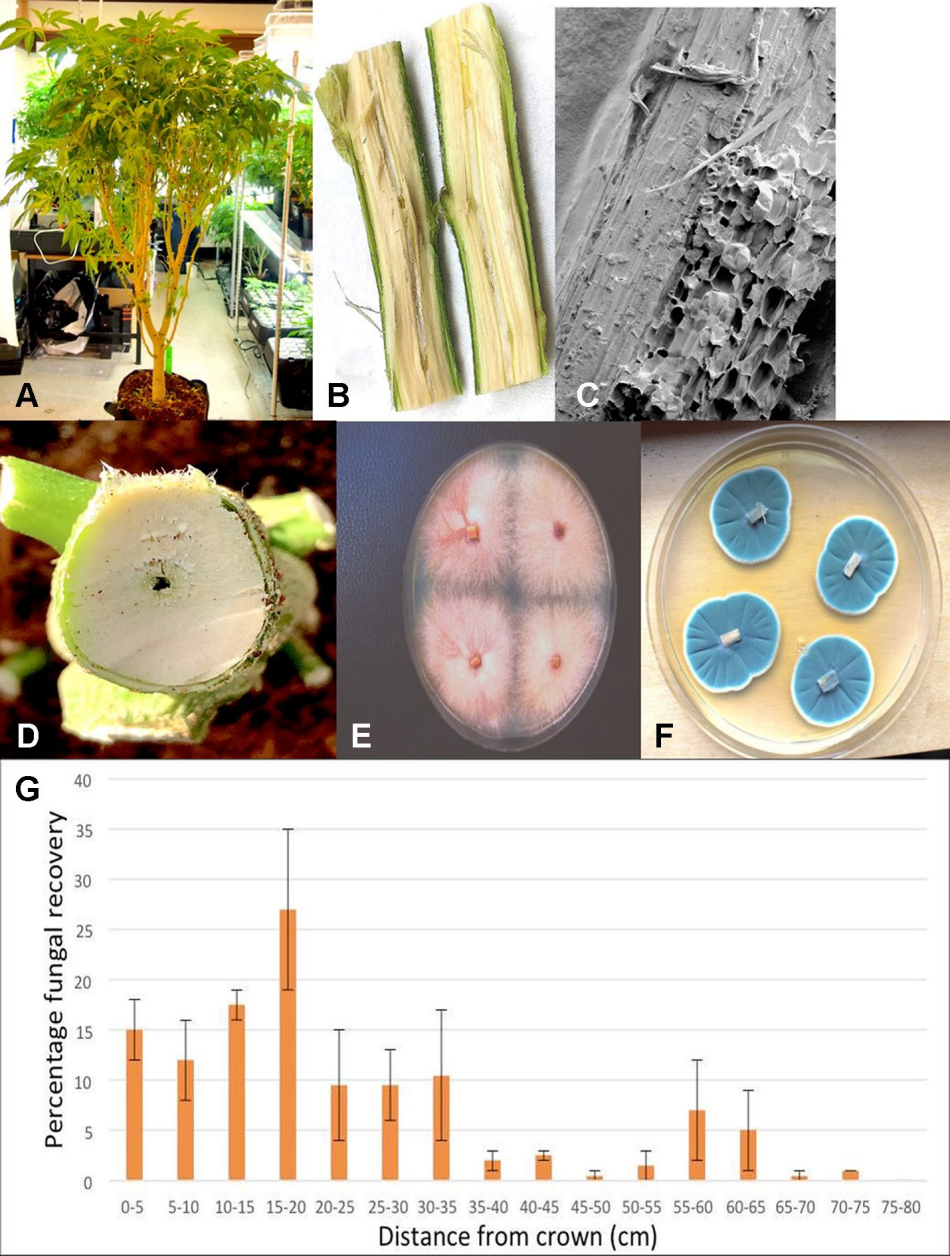
Recovery of endophytic fungi from cannabis stem tissues. **(A)** Plant grown indoors in coco substrate and used for sampling studies. **(B)** Longitudinal section though the main stem near the crown showing the central pith tissue. **(C)** Scanning electron microscopic image of the pith region showing loosely arranged parenchyma cells (arrow). **(D)** Young stem higher up the plant showing initial stages of pith development and hollow space. **(E)** Cross-section through the main stem of a cannabis plant showing the interior of the central pith which has become hollow. **(F)** Recovery of *Fusarium oxysporum* from central pith tissues near the crown region of the plant. **(G)** Recovery of *Penicillium chrysogenum* from central pith tissues near the crown region of the plant. **(H)** Frequency of recovery of total fungal species from crown and stem tissues at various distances away from the base of a cannabis plant grown in coco substrate in an indoor environment. Tissues were dissected and surface-sterilized and plated onto PDA+S. Data are from two separate experiments, representing two plants with four replicate dishes at each of 15 sampling distances. Bars show standard errors of the mean.

Plants grown in coco fiber substrate and sampled for presence of fungi in the pith and cortical/vascular tissues, as well as petiole and nodal segments, showed the presence of many fungal species, including *C. globosum*, *T. (Polyporus) versicolor*, *T. harzianum* (**Figure 14A**), *F. oxysporum\* (**Figure 11F**), and *P. chrysogenum* (**Figure 11G**). In addition, a low frequency of *Lecanicillium lanosoniveum* and a *Simplicillium* sp. were recovered from nodal segments (**Figure 14A**). The overall frequency of isolation of these endophytic fungi was greater in tissues sampled near the crown of the plant and was reduced progressing upward to a distance of 30–35 cm; following that, the incidence of recovery of these fungi was sporadic (**Figure 11H**). From surface-sterilized stem, petiole and nodal segments, recovery of *Penicillium* species (identified as *P. olsonii* and *Penicillium griseofulvum*) was high and was seen to be emerging from the cut ends (**Figure 14B**), and spore production was observed internally within pith tissues and adjacent to pith cells (**Figures 14C, D**).

### Endophytic Colonization of Stem Tissues

**Figure 12:**
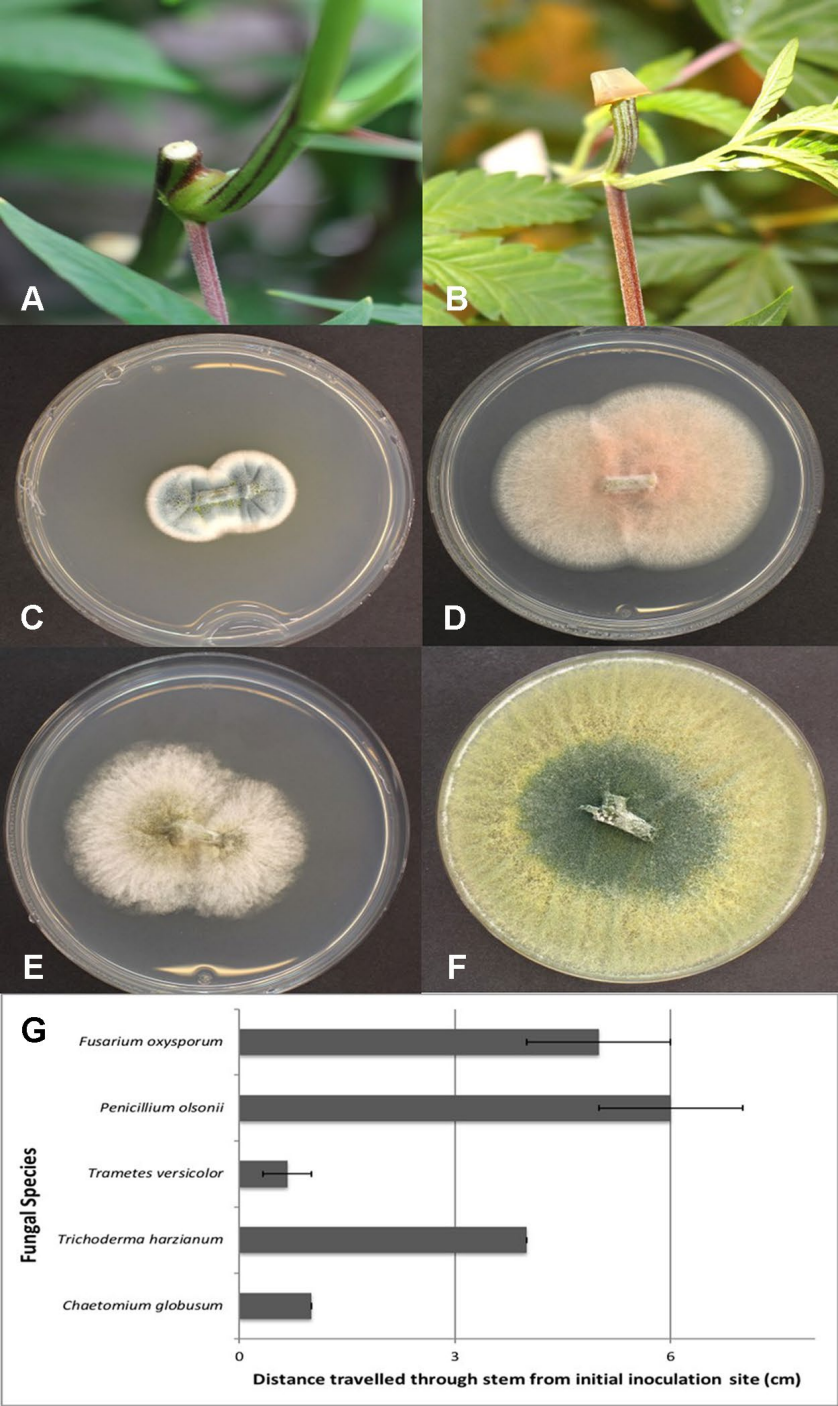
Assessing the extent of colonization of endophytic fungi inoculated onto exposed stem surfaces of *Cannabis sativa*. **(A)** Cut surface of stem following pruning. These cuts were made at the top of the plant, 65 cm from the crown region. **(B)** Inoculation method used to assess extent of colonization by placing a mycelial plug at the end of the cut stem. **(C)** Recovery of *Penicillium olsonii* from colonized stem. **(D)** Recovery of *Fusarium oxysporum* from colonized stem. **(E)** Recovery of *Chaetomium globosum* from colonized stem. **(F)** Recovery of *Trichoderma harzianum* from colonized stem. **(G)** Distance travelled downward through stem 7 days following inoculation with the fungi shown in **(C–F)**. All tissues were surface-sterilized prior to plating onto PDA+S. Data are from eight replications in each of two experiments. Bars show standard errors.

Mycelial plugs of five of the endophytic fungi recovered from cannabis stem tissues, when placed on freshly exposed stem surfaces (**Figures 12A, B**), demonstrated the ability of these fungi to colonize internally for distances of up to 6 cm within 7 days. The growth of *F. oxysporum* and *P. olsonii* was the greatest, followed by *T. harzianum*, and then *C. globosum* and *P. versicolor* (**Figure 12G**). Since the tissues were surface-sterilized before plating, the fungi recovered (**Figures 12C–F**) originated from inside the stem tissues, while control tissues yielded no fungi except for occasional (less than 5%) contamination by *Penicillium* species.

### Mold Sampling on Bud Tissues

**Figure 13:**
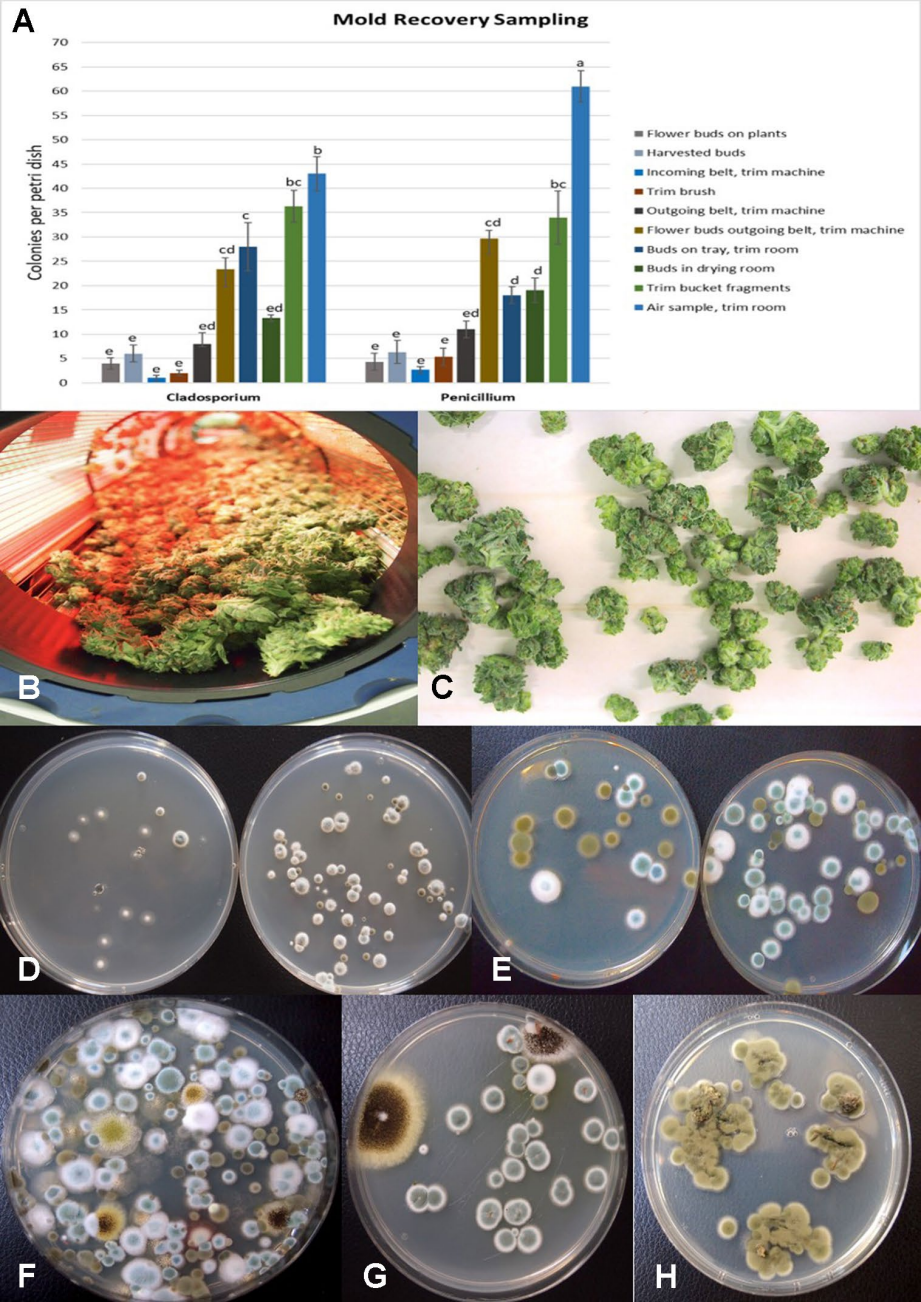
**(A)** Recovery of colony-forming units of *Cladosporium* and *Penicillium* species on potato dextrose agar at various stages of sampling of cannabis tissues, starting from buds on plants to harvested and mechanically trimmed buds. Swabs were taken of buds entering into a mechanized trim operation at different stages as indicated on the graph. Final samples were taken from trim buckets and air in the trim room, and from dried buds prior to packaging. The data are from three repeated sampling times conducted in two facilities. A minimum of eight replicate petri dishes were used at each sampling stage. Bars show +/− standard errors and were analyzed for significant differences using ANOVA. Means followed by a different letter are different according to Tukey’s HSD test at P = 0.05. **(B)** Trimmed buds leaving the trim machine. **(C)** Trimmed buds on the conveyor belt. **(D)** Recovery of *Penicillium* species from swabs taken of buds prior to being trimmed (left) compared to buds that had been trimmed (right). **(E)** Recovery of *Penicillium* and *Cladosporium* species from swabs taken of buds prior to being trimmed (left) compared to buds that had been trimmed (right). The number of *Penicillium* colonies recovered was increased following trimming. **(F)** Colonies of *Penicillium*, *Aspergillus*, and *Cladosporium* species from air samples collected from within a trim room. Dishes were left exposed for 60 min and taken back to the laboratory to allow for colony development and enumeration. **(G)** Swabs taken of indoor-grown dried cannabis buds showing growth of *Aspergillus niger* (black colonies) and *Penicillium olsonii* (blue-green colonies). **(H)** Swabs taken of dried field-grown cannabis buds and tissue segments plated on potato dextrose agar showing development of *Cladosporium westerdijkieae*.

The results from mold sampling on greenhouse-grown cannabis buds of pre- and post-harvests are shown in **Figure 13**. A low incidence (5–10 colony-forming units (cfu) per petri dish) of *Cladosporium* and *Penicillium* were found on these buds (**Figure 13A**). Following a mechanized trim operation (**Figures 13B, C**), the frequency of mold colonies increased to 25-30 cfu. The fragments of leaves and bracts that were removed from the buds after the trim and collected in a trim bucket were found to have a high mold count of up to 38 cfu present (**Figure 13A**). There was a large increase in the recovery of mold colonies, particularly those of *Penicillium*, from bud tissues before and after the trim operations (see **Figure 13A**, “harvested buds” *versus* “buds on tray.” A comparison of the colonies developing on PDA+S before and after the bud trimming operation (right petri dish in both photos) in two growing facilities is shown in **Figures 13D, E**. Petri dishes left exposed in the room where the trimming operation was being conducted showed a diverse population of mold colonies in the air, representing mostly *Penicillium* species and a few *Aspergillus* colonies were recovered (**Figure 13F**). Swabs of buds in the drying room showed the presence of *Penicillium*, *Aspergillus* and *Cladosporium* on tissues from two different production facilities (**Figure 13G**). Selected colonies were transferred to fresh PDA+S dishes for subsequent molecular confirmation of species identification using ITS1-ITS2. In sampling conducted of field-grown, harvested and dried buds of cannabis, the primary mold found to be present was *C. westerdijkieae* (75% frequency of total fungi isolated) (**Figure 13H**), and a low population of *Alternaria alternata* was also present (20% frequency) as well as some colonies of *P. olsonii* (5% frequency).

**Figure 14:**
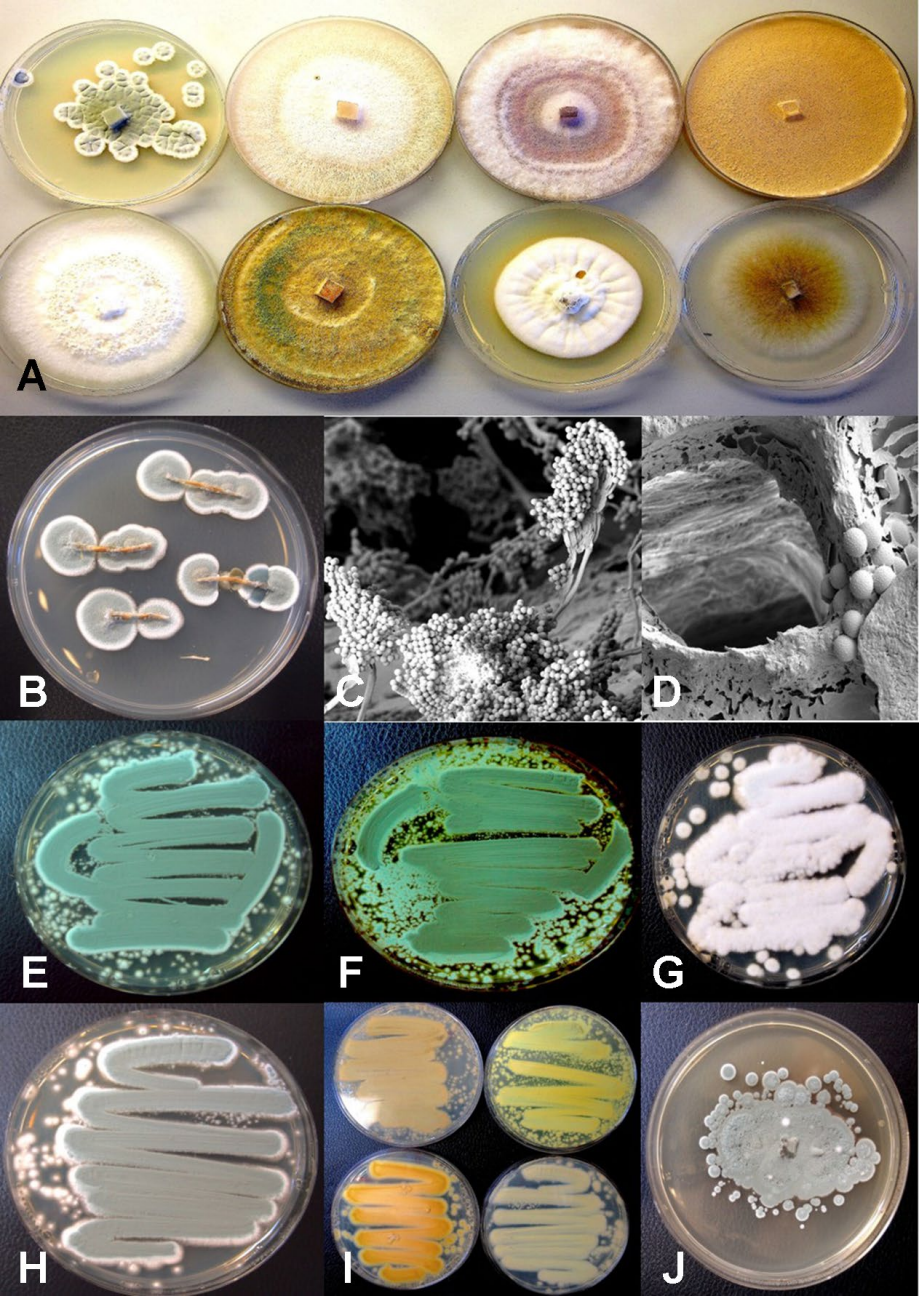
Colony morphology of endophytic fungi and contaminant fungi recovered from cannabis tissues. All colonies were grown potato dextrose agar. **(A)** Endophytic fungi recovered from surface-sterilized stems, petioles, and nodal segments after 10 days of growth in culture. From left to right (top row) are *Penicillium chrysogenum*, *Fusarium oxysporum*, *F. oxysporum*, and a *Fusarium* sp. Bottom row—*Trametes (Polyporus) versicolor*, *Trichoderma harzianum*, *Simplicillium lanosoniveum*, and *Chaetomium globosum*. **(B)** Emergence of *Penicillium olsonii* from stem pieces following surface-sterilization, indicating that internal colonization of tissues had occurred. **(C, D)** Scanning electron micrographs of dissected pith tissues from cannabis stems showing profuse sporulation of *P. olsonii* and spores adjacent to parenchyma pith cells. **(E–H)** Cultures of *Penicillium* species streaked out from swab transfers made from pure cultures originating from cannabis buds and incubated for 96 h to show colony color development. **(E)** *Penicillium spathulatum*. **(F)** *Penicillium citrinum*. **(G)** *Penicillium simplicissimum*. **(H)** *Penicillium olsonii*. **(I)** The underside of colonies of the same four *Penicillium* species after growth for 96 h. The unique colors of these species could be used for preliminary identification purposes. **(J)** Colony of *Aspergillus sydowii* after 2 weeks of growth originating from cannabis bud tissue.

Using ITS1-ITS2 rDNA sequence comparisons, up to six species of *Penicillium* were identified in the collection of isolates made from indoor air samples or those originating from cannabis bud tissues (**Table 1**). These were *Penicillium spathulatum* (**Figure 14E**), *Penicillium citrinum* (**Figure 14F**), *Penicillium simplicissimum* (**Figure 14G**), *P. olsonii* (**Figure 14H**) and *P. griseofulvum*. These colonies were subcultured by streaking a spore mass collected using a cotton swab onto PDA+S dishes where they grew and sporulated within 96 hr. The individual species produced distinct pigments in culture when viewed from below, ranging from dark gray to yellow, tan brown, and beige that facilitated identification (**Figure 14I**). To obtain an estimate of the overall frequency of recovery, from a total of 124 isolates of *Penicillium* species subcultured in this study, 48 (38%) was *P. spathulatum*, 22 (17%) was *P. citrinum*, while *P. simplicissimum* and *P. olsonii* were recovered at 20 and 21% each, respectively. A low recovery of *P. griseofulvum* and *Penicillium sclerotiorum* was also recorded (2% each).

**Table 1:** Occurrence of fungal species on indoor-grown cannabis inflorescences (pre-harvest and post-harvest buds) and in air samples. Where present, samples showing occurrence of these fungal species on outdoor samples is marked (*).

Fungal species^a^ (GenBank Accession No.)	On cannabis buds^b^ attached to plant	On harvested and^b^ trimmed cannabis buds	In air samples^c^
Alternaria alternata* (MK106666)	+	+	+
*Beauveria bassiana* (MK106662)	+	+	+
*Botrytis cinerea* (MH782039)	+	+	+
Cladosporium westeerdijkieae* (MK106665)	+	+	+
*Fusarium oxysporum* (MH782043)	–	–	+
*Penicillium citrinum*	–	+	+
*Penicillium copticola* (MH782038)	+	+	–
*Penicillium corylophilum* MK106659	–	–	+
*Penicillium griseofulvum* (MN133842)	–	+	+
*Penicillium olsonii* (MH782040)	+	+	+
*Penicillium sclerotiorum* (MN133846)	–	+	–
*Penicillium simplicissimum*	+	+	+
*Penicillium spathulatum* (MK106664)	+	+	+

a Species identification was made following PCR of the ITS1-5.8S-ITS2 region of ribosomal DNA and comparisons of sequence identity in GenBank. Only values > 99% were used.

b Fungal occurrence was determined from swabs of the bud surface made using cotton swabs and streaking onto potato dextrose agar containing 100 mg/L streptomycin sulphate. Data are from a minimum of 5 replicates and the sampling was conducted at three different times.

c Colony-forming units were recorded on Petri dishes containing potato dextrose agar plus 100 mg/streptomycin sulphate that were exposed for 60 min to the ambient environment of a greenhouse facility or an indoor facility used to grow cannabis plants.

d Absence (–) or presence (+) of the respective species was recorded after 5-7 days of incubation under ambient laboratory conditions (21-24 C).

^*^Also detected on outdoor grown cannabis bud tissues.

## Discussion

Pathogenic fungi that cause diseases, as well as molds that affect cannabis growth and quality, are documented in this study. Molds are defined as fungi present on living or dead plant materials that are not associated with disease symptoms and may be present as incidental contaminants in the air or on growing substrates, or be part of the succession of microbes that decompose plant materials. These pathogens and molds were found to occur on cannabis plants during cultivation in greenhouse and indoor controlled environment growing facilities in British Columbia as well as in Ontario, as well as in outdoor field environments. There is a scarcity of previous research on this topic, and many of the fungi and molds described here are previously unreported from cannabis. In addition, we describe the presence of endophytic fungi (those that occur internally within plant tissues without causing any apparent symptoms). No apparent disease symptoms that could be ascribed to bacterial or viral infections were noted in this study.

McPartland (1991, 1992, 1994) identified a range of plant pathogens and molds that affect cannabis during production, and recent research has described the use of molecular-based culture-independent approaches to detect molds that occur on dried cannabis products (McKernan et al., 2016a; McKernan et al., 2016b; Thompson et al., 2017). Additional research has described the occurrence of a range of culturable fungal and bacterial species that inhabit cannabis and hemp tissues internally (Gautam et al., 2013; Kusari et al., 2013; Scott et al., 2018). These previous studies demonstrate the broad diversity of microbes that can be present on, or associated with, cannabis tissues; some of which may be beneficial and others detrimental to plant growth. Our results confirm the occurrence of a range of pathogens and molds on cannabis plants and furthermore identify the potential origins and spread of these microbes within different growing environments.

On root systems of cannabis plants, pathogens that included species of *Fusarium* and *Pythium* caused browning and decay on roots that resulted in stunted growth, yellowing, and sometimes death of the affected plants. Up to four species of *Pythium* and three of *Fusarium* were identified. One new species reported here (*P. catenulatum*) was recovered at a low frequency (4% of total isolates). While this species has been shown to cause root rot on soybean and corn seedlings (Dorrance et al., 2004), its pathogenicity on cannabis plants awaits confirmation. The potential sources of inoculum of these pathogens include contaminated growing substrates, diseased cuttings, and air-borne or water-borne propagules, as well as residual inoculum from previous crops. Reproduction of these pathogens on diseased tissues can further add to the inoculum load and lead to further spread within a cannabis growing facility. Sanitization methods to ensure that introduction and spread of pathogens within a cannabis growing facility are minimized are needed. Foliar pathogens such as powdery mildew and *Botrytis* bud rot can similarly spread as air-borne inoculum or through vegetative propagation. Both of these pathogens are known to reduce growth and quality of cannabis plants, and disease management is difficult. In the case of *Botrytis*, infection of inflorescences during production can lead to significant post-harvest losses during storage. A recent review describes approaches to management of diseases caused by *B. cinerea* (AbuQamar et al., 2017). Monitoring studies on pathogen and mold spore levels within cannabis growing facilities would provide useful insights into the diversity and changes that occur in these populations.

In the present study, repeated monitoring studies were conducted in an indoor growing environment and a greenhouse environment over a 6-week and 4-week period, respectively. We observed that indoor growing facilities harbor a range of air-borne *Penicillium* species, as well as *Cladosporium* (identified as *C. westerdijkieae*, formerly *Cladosporium cladosporioides*) (Bensch et al., 2018) and overall population levels were lower compared to a greenhouse growing environment, which had higher levels of *Cladosporium*. The populations of the different fungi detected in the indoor growing facility varied over time, and there was no consistent trend observed. Applications of the biocontrol products RootShield (containing *T. harzianum*) and BotaniGard (containing *B. bassiana*) was shown to result in air-borne spread as detected on the PDA+S dishes in the weeks following application. From field-grown bud samples, the primary mold identified was *Cladosporium*, followed by *Alternaria*, which are predominant molds found outdoors during the summer (Ren et al., 1999; de Ana et al., 2006).

There are likely to be seasonal differences in the occurrence of these air-borne contaminants (Kuo and Li, 1994; de Ana et al., 2006; Khan and Wilson, 2003). On industrial hemp plants grown under field conditions, higher frequencies of fungi were present during the month of July compared to June or August (Scott et al., 2018). Highest proportions of fungi were recovered from hemp leaf tissues compared to petioles and seeds and included *Alternaria* and *Cladosporium* in addition to other genera (Scott et al., 2018). In contrast to the findings of previous researchers (Gautam et al., 2013; Kusari et al., 2013) and those reported in the present study, however, no species of *Penicillium* were recovered from field-grown hemp tissues (Scott et al., 2018). This could potentially reflect a difference between indoor and outdoor growing environments with regard to microbial communities, or differences between marijuana and hemp plants in their endophytic composition. Thompson et al. (2017) reported the following genera, in decreasing intensity of detection, to be present on cannabis buds obtained from dispensaries in northern California (growing environments were not specified): *Penicillium*, *Cladosporium*, *Golovinomyces*, *Aspergillus*, *Alternaria*, *Botryotinia*, *Chaetomium*, and a low frequency of *Fusarium* (Thompson et al., 2017). Most of these fungi are common constituents of outdoor and indoor air samples (Meklin et al., 2007), and all of them were identified in the present study to occur on cannabis tissues to varying extents. Other studies have confirmed the presence of *Penicillium* and *Aspergillus* species as contaminants on cannabis buds (McPartland, 1994; McKernan et al., 2016a; McKernan et al., 2016b), as well as low detection of *F. oxysporum* (McKernan et al., 2016b). These molds are present in soil and on plant materials (Houbraken et al., 2010; Garba et al., 2017) and can also be found in the greenhouse environment (Gamliel et al., 1996; Katan et al., 1997; Punja et al., 2016) and in residential homes (Kuo and Li, 1994; Ren et al., 1999; de Ana et al., 2006). Surprisingly, the overall recovery of *Aspergillus* species on potato dextrose agar in the present study was low (less than 1% of the total fungi quantified). This could reflect their lower overall numbers, or the difficulty in recovery of this genus which has been reported to grow slowly on many culture media (McKernan et al., 2016a). Two species were recovered in this study, *Aspergillus sydowii* and *A. terreus*, which grew slowly in culture on PDA (**Figure 14J**). Both species can be found in soil and can contaminate food products, and *A. terreus* has been reported to be an endophyte (Waqas et al., 2015).

The occurrence of a broad diversity of fungi, some of which are potential plant pathogens, in unsterilized coco fiber commonly used as a substrate for growing cannabis plants, was demonstrated in this study. Coco is produced from the processing of coconut husks that are grown primarily in tropical climates and then dried and bagged for export. Methods for sterilization of coco products (if used) are not always stated, and if conducted, may be ineffective at eliminating the vast diversity of fungi that are naturally associated with the progressive celluloytic decomposition of this plant material. Fungi present in coco fiber, and consequently that could end up colonizing cannabis plant tissues, included *C. globosum*, *P. chrysogenum* and *P. olsonii*, *A. niger*, *T. harzianum*, *T. versicolor*, *B. bassiana*, as well as species of *Simplicillium* and *Lecanicillium* (*Akanthomyces*). Gautam et al. (2013) recovered *P. chrysogenum* and *A. niger* from cannabis leaf, stem, and petiole tissues from field-grown plants. Both *B. bassiana* and *T. harzianum* are known to have endophytic activity (Ownley et al., 2008; Ownley et al., 2010; Taribuka et al., 2017; Vega, 2018). *Trametes versicolor* is a widely distributed wood decomposing Basidiomycete and a secondary plant pathogen, while *Simplicillium* and *Lecanicillium* are both entomopathogens and endophytes (Gurulingappa et al., 2011; Lim et al., 2014; Vega, 2018). *Chaetomium globosum* is commonly found in indoor environments (Wang et al., 2017). The recovery of such a broad range of fungi from cannabis plants grown in coco fiber in an indoor environment indicates propagules of these fungi that were likely to have been present in the coco growing medium.

Endophytic colonization of cannabis stem tissues, and the progression of internal colonization from the crown region to upper portions of the plant, by some of the fungi recovered from surface-sterilized leaf, petiole, and axillary buds, was demonstrated in this study. The occurrence of endophytic fungi, as well as a broad range of bacterial species, has been previously reported in cannabis and industrial hemp tissues (Gautam et al., 2013; Kusari et al., 2013; Scott et al., 2018) as well as in many other plant species (Bamisile et al., 2018). Our findings indicate that the growing substrate can harbor fungi (as well as a wide range of bacteria, which were not quantified) and movement through the plant from the roots and crown tissues into the pith tissues can distribute the microbes. The pith of plants consists of loosely organized spongy parenchyma cells which store and transport water and nutrients (Fujimoto et al., 2018). In cannabis plants, the pith also disintegrates to produce a hollow central core (see **Figure 11E**) that can allow for movement of mycelium and spores, as well as bacterial cells, readily up through the plant. Spores of *Penicillium* were observed to be present in the pith tissues. As well, the potential for colonization of exposed stem surfaces following pruning, followed by internal colonization of the stem, presumably also through the pith tissues into the plant, was demonstrated. Whether this mode of infection can result in transmission of pathogens through vegetative cuttings used for propagation or not remains to be confirmed. The occurrence of damping off symptoms (see **Figures 3D, E**) associated with *F. oxysporum* on stem cuttings suggests that spread from the pith tissues may have taken place.

Epiphytic colonization from spores of common aerially dispersed fungi such as *Cladosporium* and *Penicillium* onto cannabis tissues is also an important source of mold contamination. In particular, mature inflorescences that secrete resinous compounds from glandular trichomes (Andre et al., 2016) are exposed to pre- and post-harvest contamination by airborne spores that are deposited and adhere to the sticky surface, as demonstrated through scanning electron microscopic observations in this study. Furthermore, colonization of cut and exposed stem surfaces during pruning practices can allow entry of these fungi and their potential establishment as endophytes in cannabis plants, as previously discussed. Previous studies have associated endophytic colonization of cannabis tissues by bacteria and fungi with potentially beneficial effects on the plant, such as protection against diseases, enhancement of plant growth, increased uptake of nutrients, etc. (Gautam et al., 2013; Kusari et al., 2013; Scott et al., 2018). However, there are no studies confirming the *in situ* benefits to cannabis plants attributable to these endophytes. The proposed antagonism to pathogens has been based solely on *in vitro* antagonism experiments (Gautam et al., 2013; Kusari et al., 2013; Scott et al., 2018) and their ability to produce anti-fungal compounds (Scott et al., 2018) or zones of inhibition on agar media (Gautam et al., 2013; Kusari et al., 2013). As stated by Schulz and Boyle (2006) “Endophytes represent, both as individuals and collectively, a continuum of mostly variable associations: mutualism, commensalism, latent pathogenicity, and exploitation.” This includes saprophytes growing on dead or senescent tissues after an endophytic growth phase in the plant, avirulent microorganisms, latent pathogens, virulent pathogens in the early stages of infection, as well as beneficial microbes (Schulz and Boyle, 2006). Additional studies are required to confirm at which point in the spectrum of these interactions the endophytes reported in cannabis plants may exert beneficial/detrimental effects on growth and quality of the plants.

In forest tree species, endophytic fungal species are commonly present and can remain latent until environmental conditions cause them to become pathogens (Arnold, 2007; Sieber, 2007). Therefore, their beneficial or mutualistic roles can remain inconclusive. Not all endophytes can be assumed to be beneficial through their association with, and recovery from, internal tissues of cannabis plants or because they produce anti-microbial compounds *in vitro*. Our findings suggest that a large proportion of fungal endophytes of cannabis arise as contaminants originating from the growing medium or the external environment. Many of the fungi can impart negative consequences to the plant—they can inhabit the pith tissues and cause discoloration, they may end up on the inflorescences and result in higher mold counts, or they can interfere with vegetative propagation of the plant through cuttings or using tissue culture micropropagation (authors, unpublished observations). Some of the genera reported to be endophytic e.g., *Penicillium* and *Aspergillus* (Gautam et al., 2013; Kusari et al., 2013) are also mycotoxin producers (Abbott, 2002; McKernan et al., 2016a; Thompson et al., 2017; Perrone and Susca, 2017). They have also been associated with asthmatic and allergic conditions when present in high numbers in indoor environments (Ren et al., 1999; de Ana et al., 2006). *Cladosporium* may also produce mycotoxins (Alwatban et al., 2014) and contribute to the indoor mycoflora associated with asthmatic conditions and is commonly found on plant materials and in indoor environments (Bensch et al., 2018). Therefore, a detailed analysis of the potential negative effects of endophytic fungi on growth and quality of cannabis plants is required.

The most prevalent *Penicillium* species recovered in the present study from cannabis bud tissues and indoor air samples was *P. spathulatum*, followed by *P. simplicissimum* and *P. citrinum*. In a previous study, *Penicillium copticola* was isolated at a high frequency from the twigs, leaves, and apical and lateral buds of cannabis plants (Kusari et al., 2013), and *P. olsonii* was isolated from cannabis stems and buds (Punja, 2018). These species of *Penicillium* are reported to occur as indoor molds (*P. spathulatum*, *P. citrinum*), are found on decaying vegetation (*P. simplicissimum*, *P. olsonii*), and occur as contaminants of food and feedstuff (*P. spathulatum*, *P. simplicissimum*, *P. citrinum*). *Penicillium spathulatum* is present in indoor environments and is also found in soil and on food and feedstuff and occurs as an endophyte (Frisvad et al., 2013). It was reported to produce the anticancer compound asperphenamate. *Penicillium simplicissimum* occurs as a contaminant in food and is commonly found in decaying vegetation and produces a range of mycotoxins in culture. It is also reported to occur as an endophyte and promotes plant growth (Hossain et al., 2007). *Penicillium citrinum* has a worldwide distribution and has been isolated from various substrates such as tropical soil, cereals, spices, and indoor environments (Samson et al., 2004), and it is reported to be an endophyte and promotes plant growth (Khan et al., 2008;Houbraken et al., 2010; Waqas et al., 2015). Citrinin, a nephrotoxin mycotoxin named after *P. citrinum*, is produced by *P. citrinum*. *Penicillium olsonii* is found in decaying vegetation, soil and on foods and causes a post-harvest fruit rot of tomato (Chatterton et al., 2012; Anjum et al., 2018); it was the main *Penicillium* species recovered from field-grown dried cannabis buds in this study. *Penicillium chrysogenum* was isolated from pith tissues in the current study and has a worldwide distribution but is commonly found in indoor environments, especially in damp locations (Samson et al., 2010; Andersen et al., 2011). The species is most well known for its production of the antibiotic penicillin (Samson et al., 1977). *Penicillium griseofulvum* (syn. *Penicillium patulum*) has been shown to cause blue mold disease on apples (Spadaro et al., 2011) and has been isolated from other fruit species and various environments such as desert soil, cereal grains, and animal feed. *Penicillium griseofulvum* is able to produce the mycotoxins patulin and roquefortine C. Considering that *P. griseofulvum* is frequently isolated from apple, corn, wheat, barley, flour, walnuts, and from meat products, it could be a potential source of roquefortine C in food (Frisvad and Samson, 2004). *Penicillium griseofulvum* is known to also produce a useful secondary metabolite griseofulvin. Besides its recognized antifungal properties against a wide variety of plant pathogens, griseofulvin has been used for many years in medical and veterinary applications. Finally, *Penicillium corylophilum* was present in air samples but was not detected on cannabis tissues. It is not known to what extent that, if any, various secondary metabolites (extrolites) produced by these *Penicillium* species in culture are also produced in harvested cannabis buds or stems and leaves harboring these fungi. The longevity of spores of *Penicillium* and *Cladosporium* species following deposition on cannabis bud tissues is unknown.

The process of mechanical trimming of cannabis buds after harvest (wet trim) and the associated wounding of the tissues caused an observable increase in the recovery of *Penicillium* and *Cladosporium* colonies compared to untrimmed harvested buds, indicating their populations on the surface of tissues were increased. Wounding is known to increase the colonization of a range of fruits by *Penicillium* after harvest (Kavanagh and Wood, 1967; Vilanova et al., 2014). Exudation of nutrients from cut tissues would have enhanced the proliferation of these opportunistic molds. In addition, internally borne mold spores e.g., in the pith could have been released through wounding of tissues and become air-borne. *Cladosporium* is commonly found in indoor environments (Bensch et al., 2018) and was the most commonly identified mold, especially in the summer (Ren et al., 1999; de Ana et al., 2006). It was found on field-grown cannabis buds in this study, together with *Alternaria*. Internal growth and sporulation of *Penicillium* species within cannabis stem tissues and damage during harvest could also release spores that could subsequently contaminate bud tissues. Management of these molds on cannabis buds would require careful handling and drying and storage under conditions that discourage their further proliferation. The fact that they are so ubiquitous outdoors and indoors, and are prolific spore producers, as well as are harbored internally, provides additional challenges to producers aiming to achieve a high-quality, minimally contaminated product.

## Conclusion

The results from this study illustrate the challenges facing cannabis producers with regard to management of diseases and molds found on plants grown in different production environments. Air-borne saprophytic molds that end up on cannabis inflorescences as contaminants primarily include *Cladosporium* and several different *Penicillium* species. In addition, *Botrytis* bud rot can pose challenges to producers during production and also as a post-harvest problem. Most of the root-infecting pathogens are not visibly detrimental to plant growth unless infection occurs early; however, destruction of roots can result in as-yet undetermined reductions in yield and quality. Powdery mildew infection is commonly present in most production facilities and will require proactive management methods and potential identification and utility of disease-resistant genetic selections. The identification of diseases and molds of cannabis in the present study should foster additional research into their epidemiology and management. The response of different cannabis strains (genotypes) to the various pathogens identified in the current study is an important aspect of disease management, but at present, there is no published information on this topic, which will require additional research to be conducted in order to provide cannabis producers with additional approaches to pathogen reduction.

## Author Contributions

ZP formulated the concept of the project and designed the experiments, supervised the project and wrote the manuscript and prepared the figures. DC, CS, SL and JH performed the experiments and data analysis. DS performed the scanning electron microscopy. All authors discussed the results and edited the manuscript.

## Funding

Funding was provided through an industrial financial contribution from Agrima Botanicals and a Collaborative Research and Development (CRD) Grant from the Natural Sciences and Engineering Research Council of Canada (NSERC). Additional funding was provided from the B.C. Ministry of Agriculture and Agriculture and Agri-Food Canada through the Canadian Agricultural Partnership (CAP) Program.

## Conflict of Interest Statement

The authors declare that the research was conducted in the absence of any commercial or financial relationships that could be construed as a potential conflict of interest.
